# Different contributions of YAP1 and TAZ in the regulation of GIST tumorigenic properties

**DOI:** 10.1186/s12964-026-02859-3

**Published:** 2026-04-24

**Authors:** Irène Pezzati, Thania Hammoum, Clémentine Ayélé Teko-Agbo, Karidia Konate, Mathieu Beranger, Eric Vivès, Frédéric Chibon, Sandrine Faure, Pascal de Santa Barbara, César Serrano, Sébastien Deshayes, Prisca Boisguérin

**Affiliations:** 1 PhyMedExp, University of Montpellier, INSERM, CNRS, Montpellier, France; 2 Sarcoma Translational Research Laboratory, Vall d’Hebron Institute of Oncology, Barcelona, Spain; 3 Oncosarc, INSERM UMR1037, University of Toulouse, CNRS, Inserm, CRCT, Toulouse, France; 4 Department of Pathology, Oncopole Claudius Régaud, IUCT‐Oncopole, Toulouse, France

**Keywords:** GIST, YAP1, TAZ, siRNA, WRAP5, Nanoparticle, CYR61

## Abstract

**Background:**

Gastrointestinal stromal tumors (GIST) are mainly caused by gain-of-function mutations in KIT or PDGFRA genes and constitute the most common malignant neoplasm of mesenchymal origin. Dysregulation of the Hippo pathway and its downstream effectors YAP1 and TAZ has been implicated in GIST progression, yet their individual contributions remain unclear. Given emerging evidence of non‑redundant YAP1/TAZ functions in cancer, we sought to dissect their respective roles in GIST tumorigenic properties.

**Methods:**

We developed sequence‑specific siRNAs targeting YAP1 or TAZ, individually or in combination, and delivered them into GIST-T1, GIST‑430, and GIST‑882 cells using WRAP5 peptide‑based nanoparticles. Silencing efficiency and specificity were confirmed by Western blot, RT-qPCR, and immunofluorescence. Functional assays, including wound healing, Transwell migration, MTT metabolic activity, and cell counting, were used to evaluate proliferation and migration. Downstream transcriptional targets (CYR61, CTGF) and signaling proteins (KIT/AKT/ERK pathways) were quantified. Clinical relevance was assessed using progression‑free survival and metastasis data from the ATGsarc GIST cohort.

**Results:**

WRAP5 nanoparticles enabled specific and efficient knockdown of YAP1, TAZ, or both, with no cytotoxicity. Across GIST cell lines, TAZ silencing consistently reduced migration and proliferation, whereas YAP1 knockdown had minimal or cell-line-dependent effects. TAZ depletion markedly downregulated CYR61 and CTGF expression, while YAP1 silencing showed limited impact. CYR61 knockdown impaired proliferation and migration in all models, identifying it as a key mediator of TAZ-driven oncogenicity. Clinical analysis confirmed that high TAZ and high CYR61 expression were associated with shorter disease-free survival and metastasis.

**Conclusion:**

TAZ, more than YAP1, acts as a major regulator of GIST proliferation and migration through transcriptional control of CYR61. These findings highlight TAZ as a promising therapeutic target and demonstrate the utility of WRAP5-based nanoparticles for selective gene silencing in GIST.

**Supplementary Information:**

The online version contains supplementary material available at 10.1186/s12964-026-02859-3.

## Background

Gastrointestinal stromal tumors (GIST) are the most prevalent mesenchymal cancers of the digestive tract, with an estimated annual incidence of 10–15 per million individuals worldwide [1, 2]. The origin of these rare sarcomas is attributed to interstitial cells of Cajal (ICC), which act as pacemakers regulating gastrointestinal motility, or related mesenchymal progenitors [3]. GIST are primarily driven by oncogenic gain-of-function mutations in receptor tyrosine kinase genes, most notably *KIT* (75–80% of cases) and, to a lesser extent, *PDGFRA* (5–10%) [4, 5]. These mutations lead to the constitutive activation of downstream signaling pathways, resulting in uncontrolled cellular proliferation and survival [6, 7]. The process of oncogenic signaling cascades downstream of KIT involves the PI3K/AKT/mTOR and RAS/MAPK/ERK pathways. These pathways play critical roles in GIST development and progression, contributing to tumor initiation, growth, and metastatic dissemination [1, 8, 9].

The introduction in the early 21st century of tyrosine kinase inhibitors (TKI), such as imatinib mesylate (IM), has significantly improved patient outcomes by effectively inhibiting KIT-downstream PI3K and MAPK pathways [10]. Nevertheless, resistance remains a major clinical challenge in advanced and metastatic GIST, as most patients experience disease progression within 18 to 24 months of treatment, due to secondary resistance mechanisms [7]. Second- and third-line TKI therapies, including sunitinib and regorafenib, have been demonstrated to induce temporary disease control [11, 12]. However, their clinical efficacy is constrained by their capacity to target only a subset of secondary *KIT* mutations and by the molecular heterogeneity of resistance [5, 6, 13]. Another limitation shared by these therapies is their common mechanism of action: they do not address the emergence of additional mutations or the broader cellular adaptations that occur during prolonged TKI exposure [1]. In certain instances, resistance to TKI treatment can result from mechanisms that entirely bypass KIT, such as the loss of KIT expression. This results in a KIT-independent GIST phenotype, which is unresponsive to TKI treatments [14–16]. Together, these limitations underscore the urgent need for alternative therapeutic strategies and a deeper understanding of the molecular pathways that drive GIST progression and resistance.

Recent studies have identified LImb eXpression 1 (LIX1) as a novel contributor of GIST progression and therapeutic resistance. LIX1 is overexpressed in aggressive and relapsed tumors, and its levels increase upon TKI resistance [17, 18]. Functionally, LIX1 promotes MAPK pathway reactivation following KIT inhibition, thereby limiting TKI efficacy. LIX1 also regulates mitochondrial function, proliferation, and tumoral lineage identity in GIST cells in KIT-dependent contexts through the Hippo pathway and its two paralogous downstream effectors, the Yes-associated protein (YAP1) and the WW domain-containing transcription regulator protein 1 (WWTR1), also known as TAZ [17–19].

The Hippo signaling pathway is a highly conserved regulator of organ size, cell proliferation, and apoptosis, playing a crucial role in tissue homeostasis [20]. When the pathway is active, its core kinase cascade phosphorylates YAP1 and TAZ in a LATS1/2‑dependent manner, retaining them in the cytoplasm [21]. Upon Hippo inactivation, YAP1 and TAZ are dephosphorylated and translocate to the nucleus, where they mainly bind TEAD transcription factors [21]. Together, YAP1/TAZ/TEAD complex drives the expression of genes involved in cell growth, migration, survival, and tissue identity in numerous organs, including the digestive tract, such as *CTGF*, *CYR61*, *AREG*,* MYC*,* MCL-1*,* BIRC5*,* AXL* [22–25]. Furthermore, in the nucleus, YAP1 and TAZ also participate in the expression of Cyclin D1 (CD1), a key cell cycle regulator of the G1/S transition implicated in tumor invasion and resistance to therapy in solid tumors [26, 27].

Focusing on GIST, recent proof-of-concept studies have shown that dysregulation of the Hippo pathway, with YAP1 and TAZ as positive regulators of CD1, can drive tumor growth and progression. These findings establish Hippo/YAP1/CD1 signaling as a key oncogenic cascade in KIT-independent IM-resistant GIST [14, 15]. In KIT-dependent GIST cells, both sensitive and resistant to IM through KIT secondary mutations, pharmacological inhibition by the Bcl-2 inhibitor, AT101, of the YAP1/TAZ-CD1 axis has been recently shown to reduce tumor growth and induce apoptosis in preclinical GIST models [28]. Moreover, pharmacological inhibition of YAP1/TAZ with verteporfin promotes ferroptosis in KIT-dependent IM-sensitive and -resistant human GIST cells. This result correlates with aggressive tumor behavior, underscoring its therapeutic potential in cases resistant to TKI [29, 30].

It has been reported that the Hippo pathway is often dysregulated in combination with hyperactivation of YAP1/TAZ in various human cancers, including solid tumors and sarcomas [31]. In these cases, dysregulation contributes to aggressive phenotypes, therapy resistance, and metastatic dissemination [32, 33]. Despite their frequent co-activation in cancers, the term “YAP1/TAZ” is often described as interchangeable transcriptional co-activators of TEAD and studied as a single unit without any distinction in functional assays [34, 35]. Yet accumulating evidence reveals fundamental differences between them, not only in their protein domain composition and transcriptional partners, but also in their patterns of expression and regulatory behavior, which vary depending on tissue context and physiological conditions [36, 37]. These distinctions call for a careful evaluation of their respective roles in cancer, especially in sarcomas [38–41].

In this study, we aimed to individually and jointly evaluate the contributions of YAP1 and TAZ in three different GIST cell types (GIST-T1, GIST-430, and GIST-882) using specifically designed small interfering RNA (siRNA). For cellular internalization, we opted for the tryptophan and arginine-rich amphipathic peptide, WRAP5, which self-assembled into peptide-based nanoparticles (PBN) in the presence of siRNA as payload [42]. Formulated at a specific molar ratio (WRAP5:siRNA = 20:1), WRAP5-based nanoparticles were selected from a performed structure-activity study as one of the lead peptides [43], which was mainly evaluated in the U87 glioblastoma cell line [42]. More recently, we have demonstrated that WRAP5 could encapsulate up to three distinct siRNA, enabling the simultaneous silencing of three different proteins within GIST cells [44]. Compared to conventional lipid-based transfection reagents, the PBN demonstrated superior safety profiles with minimal cytotoxicity in long-term assays, while maintaining high intracellular delivery efficiency [43, 45]. Its versatility has been validated across multiple cell types, both cancerous and non-cancerous, as well as in animal models (mouse and zebrafish), making it particularly suitable for studying dose-dependent gene silencing effects in therapeutic contexts, as exemplified through different protein silencing [44, 46–48].

Our study reveals that TAZ rather than YAP1 is the dominant Hippo pathway effector driving tumorigenic behaviors in GIST cells. Using highly specific siRNA-mediated silencing, we demonstrate that TAZ depletion consistently reduces cell migration and proliferation across multiple KIT‑dependent GIST models, while YAP1 silencing produces minimal or cell‑line restricted effects. Mechanistically, TAZ controls the expression of the pro‑tumorigenic factor CYR61, whose suppression mirrors the phenotypic impacts of TAZ knockdown, identifying CYR61 as a major downstream effector of TAZ in GIST. In contrast, CTGF plays a more limited role. Finally, analysis of a clinically annotated GIST cohort confirms the biological data: high TAZ and high CYR61 expression correlate with shorter disease‑free survival and the presence of metastasis, underscoring their value as potential prognostic markers and therapeutic targets.

## Methods

### Peptide

WRAP5 peptide (LLRLLRWWWRLLRLL) was synthesized at the SynBio3 platform (IBMM Montpellier), and the crude product was purified in-house following a qualitative analysis by HPLC/MS (~ 95% purity). WRAP5 stock solutions were prepared at 400 µM or 40 µM and stored at 4 °C.

### siRNA design

To design siRNA targeting the gene of interest, a systematic bioinformatics approach was performed. First, the nucleotide sequence of the target gene was retrieved from the National Center for Biotechnology Information (NCBI) database. The coding sequence (CDS) was extracted in FASTA format. Next, potential siRNA candidates were designed using the RNAxs web tool (http://rna.tbi.univie.ac.at/cgi-bin/RNAxs/RNAxs.cgi). The extracted FASTA sequence was input into RNAxs with default design options, and the maximum number of siRNA was set to five. To ensure specificity, each proposed siRNA sequence (generally 19 to 21 nucleotides long) was subjected to a BLAST (Basic Local Alignment Search Tool) search (https://blast.ncbi.nlm.nih.gov/Blast.cgi) using the nucleotide BLAST (blastn) tool. Each siRNA was analyzed for sequence specificity by evaluating identity percentages, gaps, and alignment length. Only siRNA with high specificity to the target gene, minimal off-target interactions, and no significant matches with essential receptors or proteins were considered. Finally, selected siRNAs were synthesized and purchased through Eurogentec (see Table S1). The siRNA stock solutions were prepared in RNase-free water at 200 µM or 20 µM and stored at -20 °C, as recommended by the provider. Each siRNA was tested for specificity and efficacy in targeting the gene of interest before selecting the most effective one for subsequent experiments.

### WRAP5:siRNA nanoparticle formulation 

Nanoparticles were formulated in pure water supplemented by 5% glucose (Sigma-Aldrich) by mixing equal volumes of WRAP5 and siRNA at the corresponding molar ratio (WRAP5:siRNA = 20:1) at room temperature [42]. For PBN combining two siRNA (siYAP1 + siNEG or siTAZ+siNEG or siYAP1 + siTAZ), the two siRNA were pre-mixed according to siRNA stoichiometry 1:1 as described [44].

### Dynamic light scattering (DLS)

WRAP5:siRNA nanoparticles (WRAP5 = 10 µM, siRNA = 500 nM, *R* = 20) were evaluated with a Zetasizer NanoZS (Malvern) in terms of mean particle size (Z-average diameter) of the particle distribution and the polydispersity index (PdI). All results were obtained from three independent measurements (three runs for each measurement at 25 °C) as described [42].

### Cell culture conditions

The experiments were conducted on human KIT-dependent IM-sensitive GIST cells. The GIST-T1 cell line (primary mutation in KIT exon 11 Δ560–578 in-frame deletion), from Cosmo Bio (Japan, Catalog No: PMC-GIST01C), was established from a metastatic Asian female human untreated GIST sample [49, 50]. GIST-882 (KIT exon 13 K642E mutation) [51] and GIST-430 (KIT exon 11 Δ560–576 in-frame deletion) [52] were a gift from our collaborator, Dr. C. Serrano (Sarcoma Translational Research Laboratory, Vall d’Hebron Institute of Oncology [VHIO], Barcelona, Spain).

GIST-T1 cells were grown in Dulbecco’s Modified Eagle’s Medium (DMEM) (Corning, #10-013-CV) supplemented with 10% fetal bovine serum (FBS) (Sigma-Aldrich, #F7524) and 1% penicillin-streptomycin (Sigma-Aldrich, #P4333). GIST-430 and GIST-882 cells were cultured in Iscove’s Modified Dulbecco’s Medium (IMDM) (Sigma-Aldrich, #I3390) supplemented with 15% FBS, 1% penicillin-streptomycin, and 1% L-Glutamine (Thermo Fisher Scientific, #11524456). All the cells were passaged using Gibco™ Trypsin-EDTA (0.05%), phenol red (Thermo Fisher Scientific, #25300062). All the cells were maintained in a humidified incubator with 5% CO_2_ at 37 °C. All cultures were tested monthly to be mycoplasma-free using the MycoAlert™ PLUS Mycoplasma Detection Kit (Lonza, #LT07-710). GIST-T1 cell lines were incubated with IM (STI571, Euromedex, France) at the concentrations indicated in the figure legends.

### GIST data set

Kaplan-Meier curves of progression-free survival stratified by *YAP1*, *TAZ*, *CYR61*, or *CTGF* were obtained based on a clinically annotated gene expression data set (ATGsarc) of localized, untreated GIST (*n* = 60), quantified by microarray (ArrayExpress: E-MTAB-373) [53]. For each gene, patients were divided into high and low expression groups. The cut-off for stratifications was first set at the mean expression value, a standard approach in survival analysis to minimize bias from outliers (log-rank test). In parallel, box plot analyses were performed to compare the expression levels of these four genes between GIST patients with and without metastasis, providing additional insight into their potential association with disease aggressiveness.

### Cell cytotoxicity measurement 

The cytotoxicity induced by the nanoparticles was evaluated using Cytotoxicity Detection Kit^Plus^ (LDH, Sigma-Aldrich) following the manufacturer’s instructions and as described before [42].

### Western blotting 

For Western blot assays, 300,000 GIST-T1 and 700,000 GIST-430 cells were seeded into 6-well plates (Sarstedt, #83.3920) 24 h before the experiment. For GIST-882 cells, 500,000 cells were seeded into 6-well plates 48 h before the experiment. For nanoparticle incubation, cells were treated with 800 µL of fresh, pre-warmed serum-free culture medium and 200 µL of nanoparticle solutions at the specified concentrations, or 200 µL of 5% glucose as the non-treated control condition (N.T.). After 1.5 h, 1,000 µL of the appropriate culture medium supplemented with 20% FBS for GIST-T1 (final FBS concentration = 10%) or 30% FBS for GIST-430 and GIST-882 (final FBS concentration = 15%) was added to each well, without removing the transfection reagents. The cells were incubated for an additional 48 h before being lysed for RNA extraction or Western blot analysis.

Transfected cells were lysed as described before [44]. Proteins were revealed with the appropriate primary antibody (see Table S2) in the same blocking buffer overnight at 4 °C. After three washes with TBS-0.2% Tween, membranes were incubated for 1.5 h with the corresponding HRP-conjugated secondary antibody. VINCULIN, α-ACTININ, or β-ACTIN proteins were used as loading controls depending on the experiment (see Table S2). The signal intensities of the blots were quantified using Fiji ImageJ software as described in [44].

### Confocal microscopy 

For the confocal microscopy, 150,000 GIST-T1 cells were seeded onto 18 mm cover slips in 12-well plates (Sarstedt, #83.3921). 24 h post-seeding, cells were incubated with 400 µL of fresh, pre-warmed serum-free DMEM and 100 µL of nanoparticle solutions at the specified concentrations. After 1.5 h, 500 µL of DMEM supplemented with 20% FBS was added. After transfection, cells were fixed with 2% paraformaldehyde (PFA, CliniSciences, #15714-S) and simultaneously permeabilized and blocked using a solution of PBS containing 0.1% Tween-20/4% donkey serum (SIGMA, #D9663). Afterward, cells were incubated overnight with primary antibodies, followed by a 1-hour incubation with secondary antibodies. Nuclear staining was performed using Hoechst (1 mg/mL, Sigma, #14533), and membrane actin was labeled with Phalloidin Alexa Fluor™ Plus 647 (1:800, Thermo Fisher Scientific, #A22287). To visualize YAP1 and TAZ simultaneously, a mixed antibody staining approach was used. Finally, the cells were washed with D-PBS three times and mounted on glass slides with poly-(vinyl alcohol) Mowiol™ 4–88 glycerol Tris buffer (Biovalley). Confocal images were acquired using an inverted Zeiss LSM800 microscope with an Apo 63x/1.2 W DICIII objective. To minimize fluorophore crosstalk, sequential image acquisition was applied. For each biological replicate, 2 different arbitrary positions were selected for a z-stack mode acquisition (15 images with a z-stack interval of 0.22 μm). Acquired images were analyzed using Fiji (ImageJ) software. The sum of the fluorescence intensity was performed for each z-stack projection to obtaina single image. The threshold of each sample was adjusted to quantify the mean fluorescence of the image. Mean fluorescence of YAP1 and TAZ was normalized by the mean fluorescence of the nucleus (Hoechst signal) and then adjusted to the WRAP5:siNEG condition.

### Cell proliferation assay - MTT 

For the MTT assay, 10,000 GIST-T1 cells were seeded into 96-well plates (Corning Falcon, #353072) 24 h before the experiment. Cell proliferation was assessed using the CellTiter 96^®^ Non-Radioactive Cell Proliferation Assay (Promega, #G4000), in which the luciferase-catalyzed luciferin/ATP reaction provides an indicator of cell number, based on the enzymatic conversion of MTT into a colored formazan product. The manufacturer’s protocol was followed with slight modifications. The transfection with WRAP5:siRNA (1,200 nM:60 nM) and IM treatment (200 nM, Euromedex, #TO-I031) was performed in a total volume of 100 µL per well. Cells were incubated with 80 µL of serum-free DMEM and 10 µL of nanoparticle solutions. After 1.5 h, 10 µL of pure FBS was added, without removing the transfection reagents. All experiments were conducted in quintuplicate. At 24–48 h post-transfection, 15 µL of the Dye Solution was added to each well, and the plate was incubated for 2 h. Subsequently, 100 µl of the Solubilization Solution/Stop Mix was added to each well, and the plate was incubated at room temperature for 1 h on a rocker to ensure a uniformly colored solution. Cell proliferation was measured using an Infinity M200 Pro microplate reader (Tecan Group Ltd) at 570 nm to quantify formazan formation and at 630 nm to minimize background interference from cell debris and nonspecific absorbance.

Cell proliferation was obtained using the following formulas:

$$\text{Corrected Absorbance}=\left[\left(\mathrm{Abs}_{570}\left(\mathrm{Sample}\right)-\mathrm{Abs}_{570}\left(\mathrm{Background}\right)\right)-\left(\mathrm{Abs}_{630}\left(\mathrm{Sample}\right)-\mathrm{Abs})_{630}\left(\mathrm{Background}\right)\right)\right]$$

$$\text{Cell Proliferation Index}=\frac{\text{Corrected Absorbance}\,\left(\mathrm{Sample}\right)}{\text{Mean Corrected Absorbance}\,\left(\mathrm{WRAP5}:\mathrm{siNEG}\right)}$$

This approach normalizes the absorbance readings by removing background interference and comparing sample proliferation to the WRAP5:siNEG control. The background group was defined as the absorbance of wells containing medium without cells.

### Cell proliferation assay - cell counting 

For cell proliferation analysis by cell counting, 100,000 GIST-T1 cells were seeded into 12-well plates 24 h before the experiment. The transfection of WRAP5:siRNA (800 nM:40 nM) and IM treatment (50 nM) were performed in duplicate 24 h after seeding. Transfection was performed using 400 µL of serum-free DMEM and 100 µL of nanoparticle solution. After 1.5 h, 500 µL of DMEM supplemented with 20% FBS was added. Measurements were taken at 0 h, 24 h, 48 h, and 72 h post-treatment following an established protocol. Before counting, viable cells were washed twice with DPBS 1X and incubated for 5 min with Trypsin-EDTA (0.05%) containing phenol red to detach the adherent cells. The resulting suspension was brought to a final volume of 1,000 µL by adding 800 µL of DPBS 1X. Proliferation was assessed using the CellDrop BFx automated counter (DeNovix, Wilmington, DE, USA) with the Brightfield program. A custom program optimized for GIST-T1 was used to ensure precise quantification. For each well, four independent counts were performed on 10 µL aliquots, and the average was used to determine the final cell concentration (cells/mL). To assess cell growth, counts at each time point were normalized to the mean count at t = 0 h.

### Cell migration assay - wound healing assay 

A wound healing assay was performed to assess the migratory capacity of GIST-T1 cells. To standardize the procedure, a Culture-Insert 4 Well (ibidi GmbH, #80469) was used in a 12-well plate. To minimize evaporation, 700 µL of medium was added to the area surrounding the insert. Next, 110 µL of a cell suspension containing 100,000 cells in complete DMEM was carefully added to each well of the insert. Transfection with WRAP5:siRNA (1,600 nM:80nM) was performed 24 h after seeding. The medium was gently removed, and 90 µL of serum-free medium was added, followed by 10 µL of WRAP5:siRNA nanoparticle solution. The cells were incubated for 1.5 h at 37 °C, after which 10 µL of pure serum was added to each well. After 24 h, the insert was carefully removed from each well and the well was filled with 1 mL of complete DMEM. If necessary, 1–2 washes with serum-free medium were performed to remove detached cells and debris.

Images of the wound area were captured immediately after “scratching” (= 0 h) and at 48 h (72 h post-transfection) using the EVOS^®^ LX Core microscope system (#AMEX1000, Thermo Fisher Scientific), equipped with a 3.1 megapixel color camera (2048 × 1536 pixels, 1/2-inch sensor) and a resolution of 1024 × 768 pixels. The wound area was quantified using the Wound_healing_size_tool.ijm plugin in ImageJ, with adjusted parameters: Variance 10, Threshold 25, and Saturation 0.200. The percentage of wound closure was calculated by subtracting the area at t = 0 h from the area at t = 48 h, and the results were normalized to the siNEG condition to assess cell migration.

### Cell migration assay - transwell assay 

GIST-T1 cells were seeded and incubated as described in the “Western blot” section. After the indicated treatments, the migratory ability of GIST‑T1 cells was assessed using a Transwell assay as described previously [54]. Briefly, 4 h before seeding GIST-T1 cells into Transwell chambers (8‑µm pore size for a 24-well plate, Corning, #3422), medium in the 6-well plate was replaced by serum-free medium. Before counting, cells were washed twice with DPBS 1X and incubated for 5 min with Trypsin-EDTA (0.05%) containing phenol red to detach the adherent cells. The resulting suspension was brought to a final volume of 500 µL by adding 400 µL of DPBS 1X + 5% FBS. Cells were transferred into a tube, centrifuged, resuspended in medium without serum (125 µL), and counted. 20,000 GIST‑T1 cells were seeded into the upper chamber of the transwell, while 300 µL medium + 20% FBS was added to the lower chamber. Following an incubation of 36 h, the cells were fixed with 2% PFA. Non‑migrated cells on the upper surface of the membrane were removed using a cotton swab, whereas migrated cells on the lower surface were stained with 0.5% crystal violet (in 20% EtOH/80% H_2_O, Sigma-Aldrich, #) for 10 min. After several washing steps with DPBS 1X to remove background, the cells were imaged using an EVOS^®^ LX Core microscope system (#AMEX1000, Thermo Fisher Scientific). Amount of migrated cells was quantified using ImageJ.

### Reverse transcription and quantitative polymerase chain reaction (RT-qPCR) 

GIST-T1, GIST-430, and GIST-882 cells were seeded and incubated as described in the “Western blot” section. After an incubation period of 24 h, total RNA was extracted from the cell cultures using the RNeasy^®^ Mini Kit (Qiagen, #74104), following the manufacturer’s protocol with some modifications. Specifically, cells were lysed in 230 µL of RLT buffer per well in a 6-well plate. The lysates were then processed according to the manufacturer’s instructions to obtain RNA samples. RNA quality and concentration were assessed using a NanoDrop™ One spectrophotometer (Thermo Scientific). The extracted RNA was then reverse transcribed into complementary DNA (cDNA) using 500 ng of RNA as the input material. The reverse transcription was carried out using the Verso cDNA Synthesis Kit (Thermo Scientific, #AB-1453/B), according to the manufacturer’s instructions. Following the synthesis, the cDNA was diluted in ultra-pure RNase-free water to a final concentration of 2.5 ng/µL for use in subsequent qPCR analysis. Quantitative PCR (qPCR) was performed using the LightCycler^®^ technology (Roche Diagnostics). PCR primers were designed using the LightCycler Probe Design 2.0 software for some targets, while others were sourced from the literature. Detailed primer sequences are provided in Table S3. To ensure primer specificity, each primer was verified through a BLAST search to confirm that there were no mismatches and that only the target of interest was amplified. Additionally, the efficiency of each primer was validated by performing a dilution series of cDNA ranging from 10 ng to 0.001 ng of cDNA per reaction. For each qPCR reaction, 5 ng of cDNA was added per well in a 96-well qPCR plate, in a final reaction volume of 10 µL. Reactions were carried out using the SYBR Green detection method (Roche Diagnostics, #04887352001), qPCR was conducted in technical triplicate, and standard curves were generated for each primer pair to assess amplification efficiency. Data were normalized to the reference gene *HPRT1*, and relative gene expression was calculated using the 2^−ΔΔCt method, which compares the Ct values (threshold cycle) of the target genes relative to the reference genes and the calibrator sample. All qPCR reactions were performed in a LightCycler^®^ 480 System (Roche Diagnostics, #05015243001), and the results were analyzed and presented as mean gene expression levels relative to the reference genes.

### Statistical analysis

All statistical analyses were performed using GraphPad Prism (version 10.6.1). Data are presented as mean ± standard deviation (SD) or standard error of the mean (SEM) when appropriate, based on at least three independent experiments. Statistical analysis was performed using an ordinary one-way ANOVA followed by a Dunnett’s or a Holm-Sidak’s post-test or Kruskal-Wallis followed by Dunn’s multiple comparisons dependent on the analyzed conditions. In a case of two-group comparison, an unpaired two-tailed Student’s t-test was applied. A 95% confidence interval was applied to all tests, and statistical significance (*p*-value) was defined as follows: ns (not significant) > 0.05, * <0.05, ** <0.01, *** <0.001, and **** <0.0001. In the figures, the number of independent experiments (*N*) and the number of replicates (*n*) are shown.

## Results

### Role of YAP1 and TAZ in GIST prognosis

In our previous work, we identified *LIX1* expression in GIST tissue microarrays in 79% of all GIST specimens (61/77) as well as in 79% of high-grade GIST (34/43) [17]. Moreover, *LIX1* was assessed as a negative prognostic factor in GIST, with high expression levels associated with shorter disease-free survival and increased metastatic risk. Importantly, *LIX1* was suspected to regulate GIST cell plasticity through the modulation of *YAP1* activity, suggesting a mechanistic link between these factors [17, 55].

**Fig. 1:**
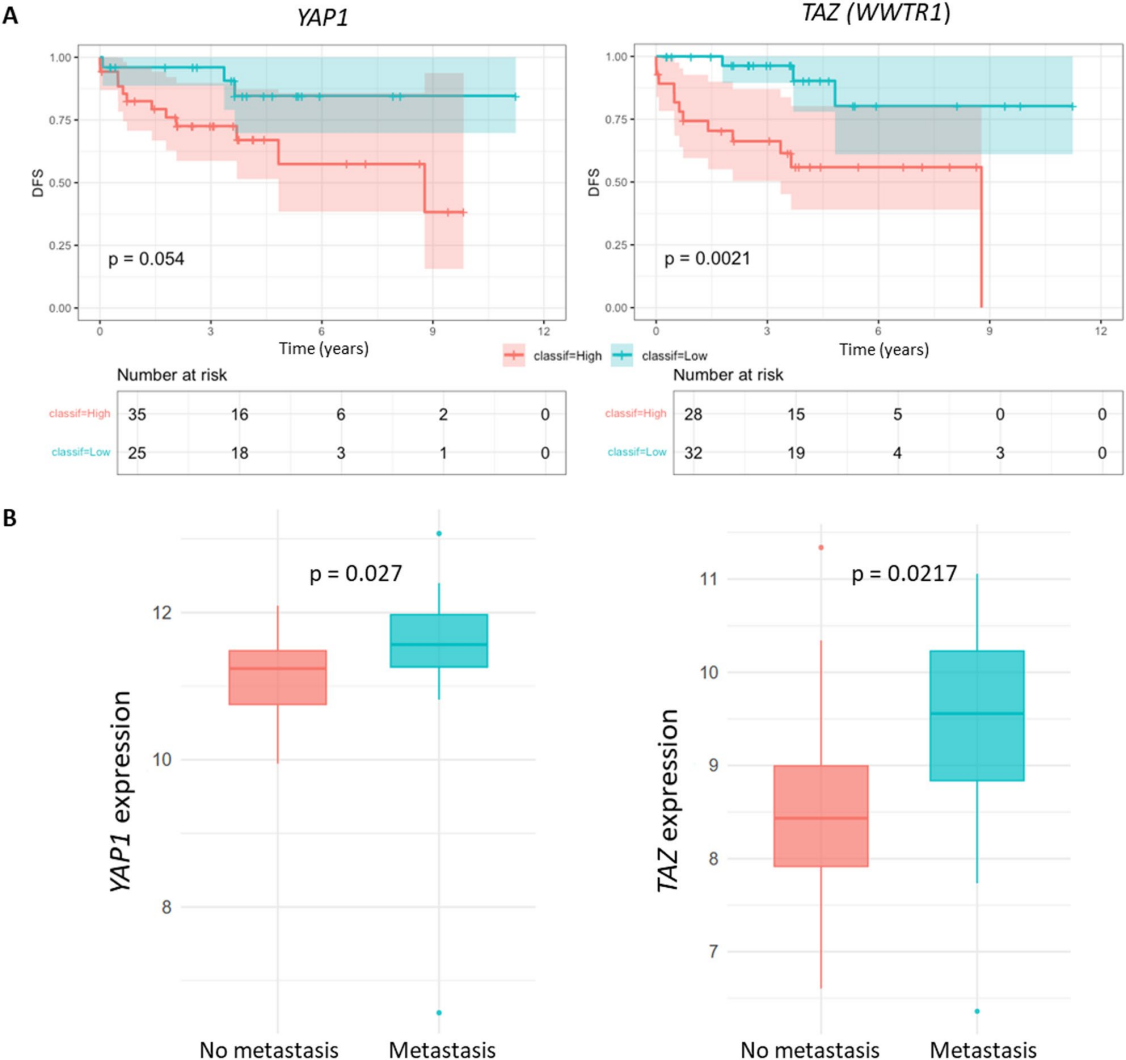
Correlation of *YAP1* and *TAZ* expression with the prognosis of GIST patients. **A** Kaplan-Meier curves of disease-free survival (DFS) from 60 total GIST patients in the ATGsarc microarray database, stratified by YAP1 or TAZ (WWTR1) expression levels. Group 1 (red): patients with high gene expression (*YAP1* = 35 and *TAZ* = 28); Group 2 (blue): patients with low gene expression (*YAP1* = 25 and *TAZ* = 32). Number at risk = number of patients at risk over time. They are summarized in the table below the curves. **B** Comparison between YAP1 or TAZ transcript levels and the presence of metastasis. *n* = 45 (red) and *n* = 15 (blue) in the no metastatic and metastatic groups, respectively. *p*-values from the log-rank test are indicated.

In the present study, we first evaluated whether the expression of *YAP1* and *TAZ* (*WWTR1*) was also associated with clinical outcome in GIST patients, as we previously did for *LIX1*. We analyzed a cohort of 60 patients from the ATGsarc microarray database, which is the only available clinically annotated, untreated, localized GIST cohort [53]. The first analyzed data set comprised 28 GIST patients with high and 32 with low *TAZ* expression, and the second one contained 35 GIST patients with high and 25 with low *YAP1* expression (Fig. 1A). Both blotted Kaplan-Meier curves revealed that a tendentially high expression of *YAP1* (*p* = 0.054) or a significantly high expression of *TAZ* (*p* = 0.0021) could be associated with shorter disease-free survival, indicating a potential role in tumor progression.

Similarly, high transcript levels of both *YAP1* and *TAZ* correlated with the presence of metastases. Both analyzed datasets comprised 15 GIST patients with and 45 without metastases (Fig. 1B). Both boxplots indicated a significant correlation of *YAP1* expression (*p* = 0.027) or *TAZ* expression (*p* = 0.0217) and metastasis formation, linking both genes to an aggressive tumor behavior.

To further prove their role in GIST, we functionally assessed their individual contributions *in vitro* experimentally.

### Specific silencing of YAP1 or TAZ using WRAP5:siRNA nanoparticles in GIST-T1 cells

To selectively investigate the individual and combined roles of YAP1 and TAZ in GIST-T1 cells, we designed for each gene different siRNA using the RNAxs web tool. Each siRNA was blasted to ensure the specificity of the sequence for its target. Two siRNA with the highest score, each targeting two different complementary regions of the targeted mRNA, were selected for evaluation (Table S1).

For the cellular internalization of the siRNA, we used the WRAP5-based nanoparticle delivery system previously validated for efficient and low-toxicity gene silencing across diverse cancerous and non-cancerous cellular models [42, 43, 46]. First, we used dynamic light scattering (DLS) to validate WRAP5:siRNA nanoparticle integrity, showing an average diameter ranging from 90 to 120 nm, with a polydispersity index (PdI) of around 0.3 (Figure S1). These relatively homogeneous populations of nanoparticles were consistent with previous reports [42, 44] and were, therefore, suitable for gene silencing experiments.

**Fig. 2:**
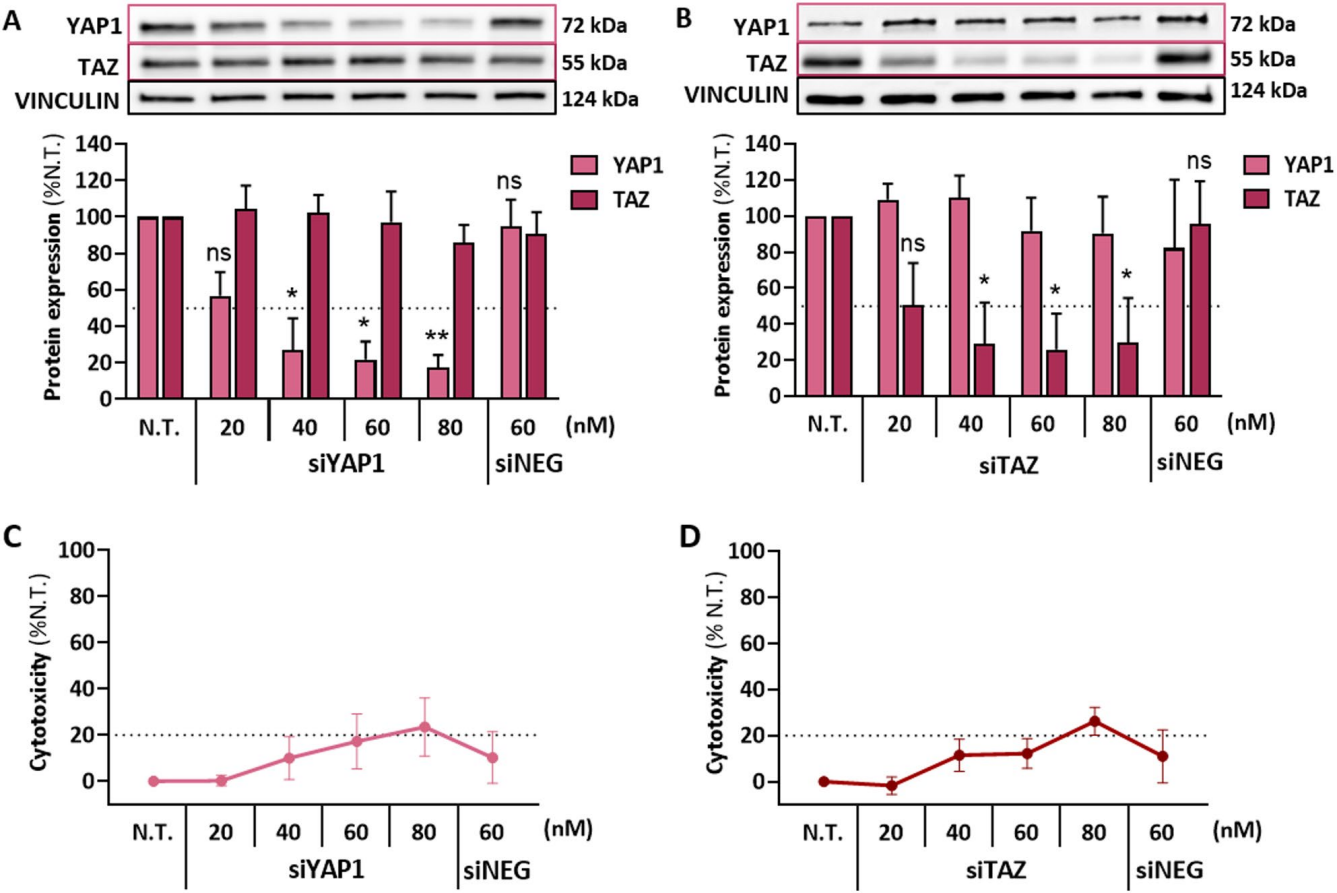
Evaluation of YAP1 or TAZ silencing in GIST-T1 cells with WRAP5:siRNA nanoparticles. WRAP5:siRNA nanoparticles delivering siYAP1 (**A**) or siTAZ (**B**) induced a dose-dependent inhibition of YAP1 or TAZ protein expression in GIST-T1 cells after 48 h of incubation, as shown by Western blot quantification. No significant toxicity was observed for siYAP1 (**C**) or siTAZ (**D**) within the tested concentration range (20 – 60 nM), as assessed by LDH assay. A slight toxicity was observed for 80 nM. Controls included untreated (N.T.) and siNEG-treated cells. Data are presented as mean ± SD for *N *= 4 individual experiments. Statistical analysis was performed using Kruskal-Wallis followed by Dunn’s multiple comparisons test versus N.T. (**A**, **B**); ns >0.05, * <0.05, ** <0.01. Analysis of TAZ expression following siYAP1 treatment and YAP1 expression following siTAZ treatment showed no significant differences compared to N.T. (data not annotated on the graph for clarity)

YAP1 and TAZ silencing were evaluated in GIST-T1 cells using WRAP5:siYAP1 and WRAP5:siTAZ in a dose-dependent manner (20 nM to 80 nM) by Western blot (Fig. 2A and B). This analysis was performed compared to non-treated GIST-T1 cells as well as to those incubated with WRAP5 nanoparticles encapsulating a siRNA without any cellular target (siNEG) to ensure that the nanoparticles do not influence cell physiology.

Concerning the YAP1 and TAZ silencing, we observed a specific knockdown of YAP1 with the WRAP5:siYAP1 nanoparticles without any effect on the TAZ protein expression (Fig. 2A**)**. The same specificity has been shown for WRAP5:siTAZ nanoparticles on the TAZ and YAP1 expression (Fig. 2B**)**. This knockdown specificity was further confirmed with additional controls: (1) alternative siRNA sequences targeting another region of the *YAP1* and *TAZ* mRNA showed no cross-silencing between YAP1 and TAZ, depending on the siRNA used (Figures S2A and S2B), and (2) WRAP5:siNEG nanoparticles did not induce any YAP1 or TAZ silencing compared to the untreated GIST-T1 cells (Fig. 2A and B, S2A, and S2B). Finally, we evaluated the persistence of YAP1 and TAZ suppression over the relevant experimental time window (up to 72 h) by Western blot (Figure S4).

For both proteins, YAP1 and TAZ, the maximal silencing efficacy reached a plateau of approximately 70% at a siRNA concentration of 60 nM, depending on the used siRNA (Fig. 2A, B, S2A, and S2B). To confirm that this range of silencing for both proteins, we encapsulated a siRNA (siYAP1TAZ) [56] that silenced both proteins (Figure S3). Using this siRNA, a silencing of around 70% for both proteins was revealed, confirming the specificity and efficacy of our designed siRNA.

 To ensure that the nanoparticles did not interfere with the cell viability, we performed for all analyzed conditions (Fig. 2A, B, S2A, and S2B) cytotoxicity assays (LDH) before cell lysis for Western blot. Indeed, we revealed minimal cytotoxicity below 20% for siRNA concentrations up to 60 nM and only some slight effects (20%-30%) using the highest siRNA concentration of 80 nM (Fig. 2C, D, S2C, and S2D). These thresholds for cytotoxicity were in the acceptable range as described in the standard ISO 10993-5 used to evaluate the in vitro cytotoxicity of medical devices [57].

Then, we compared YAP1 and TAZ expression levels depending on different percentages of GIST-T1 cell confluence (30%, 50%, 80%, and 100%) to ensure that cell confluence did not impact protein expression levels. Western blot analyses confirmed that, at working cell confluence ranges between 50% and 100%, no significant difference between YAP1 or TAZ protein expression levels was observed (Figure S5). This finding confirmed that GIST-T1 cell confluence and potent resulting contact inhibition did not affect the protein quantity of YAP1 and TAZ [58, 59].

### Simultaneous silencing of YAP1 and TAZ in GIST-T1 cells

Knowing that the WRAP5:siYAP1 and the WRAP5:siTAZ nanoparticles induce specific YAP1 or TAZ silencing (at 40 nM siRNA), we wanted to combine both siRNA to further evaluate if a synergistic silencing effect could be observed. The delivery of two or three siRNA simultaneously by the WRAP5 nanoparticles was previously described in glioblastoma U87 cells, but also in GIST-T1 cells [44]. Furthermore, as the WRAP5:siNEG nanoparticles did not affect cell physiology and cytotoxicity, we used this condition for the result normalization for all further assays.

**Fig. 3:**
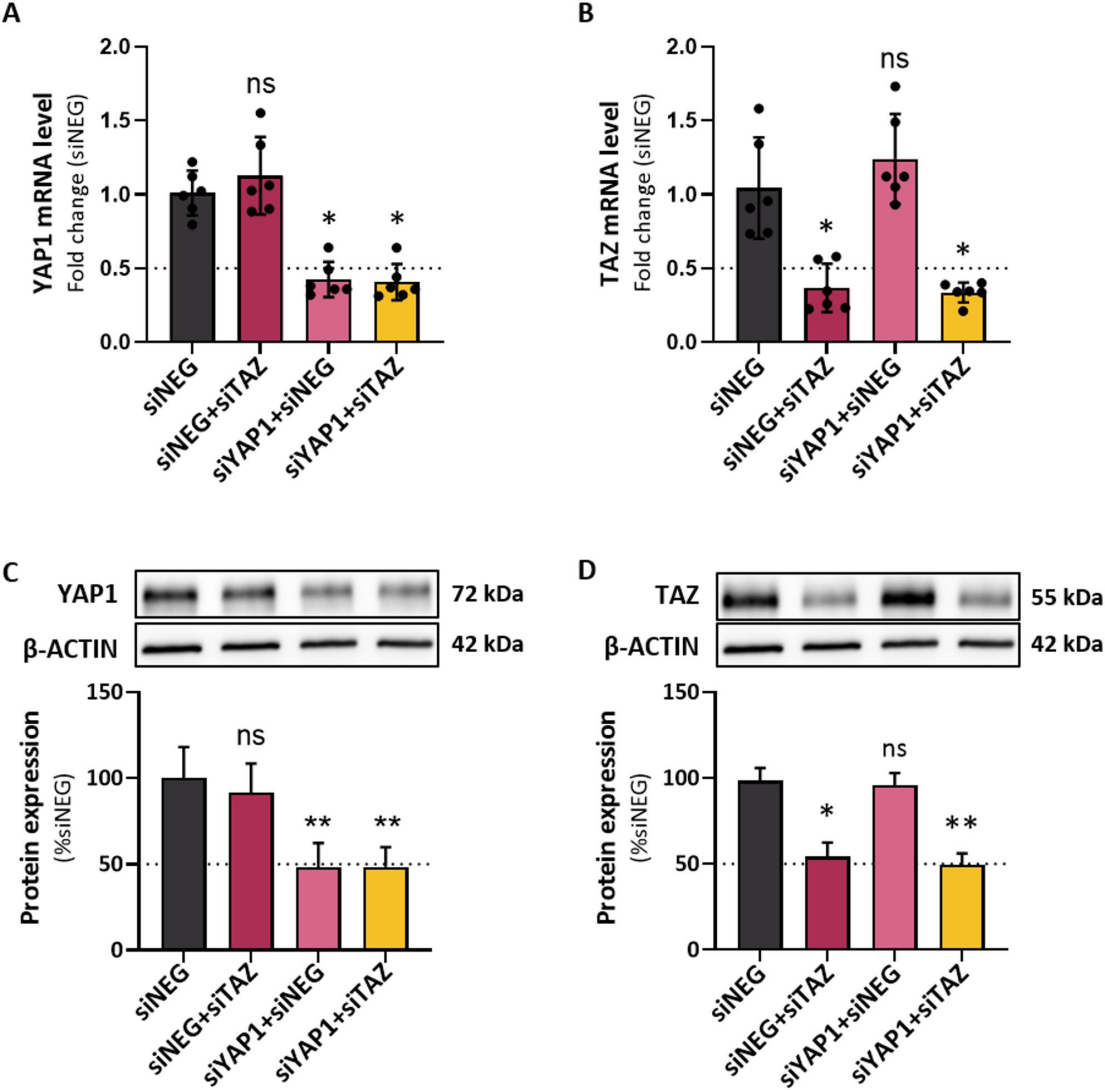
Specific targeting of YAP1 and TAZ, individually or simultaneously, using WRAP5:siRNA cocktails. WRAP5:siRNA nanoparticles delivering an equimolar cocktail of siRNAs targeting YAP1 and/or TAZ specifically inhibited YAP1 and TAZ mRNA (**A**, **B**) and protein expression levels (**C**, **D**) in GIST-T1 cells, as assessed by Western blot quantification and qPCR analysis, respectively. Conditions: WRAP5:siRNA (MR 20 with [siRNA] = 40 nM), siNEG-treated cells were used as a control condition. Data were presented as mean ± SD for *N *= 6 individual experiments. Statistical analyses were performed using Kruskal-Wallis followed by Dunn’s multiple comparisons test versus siNEG; ns >0.05, * <0.05, ** <0.01

The siRNA cocktail with an equimolar ratio (1:1) of siYAP1 and siTAZ (WRAP5:siYAP1 + siTAZ) was formulated with a constant siRNA concentration (40 nM) across conditions, meaning that [20 nM siYAP1 + 20 nM siTAZ] were used for the (WRAP5:siYAP1 + siTAZ) condition. Analyses by RT-qPCR confirmed the robust silencing (up to 70%) of *YAP1* (*p* < 0.05) and *TAZ* (*p* < 0.05) mRNA when the WRAP5 nanoparticle encapsulated both siRNA (siYAP1 + siTAZ) compared to WRAP5:siNEG (Fig. 3A and B). Comparable mRNA silencing efficiency was observed for (siYAP1 + siNEG) or (siNEG+siTAZ), allowing us to rule out any cross-talk due to simultaneous knockdown of YAP1 and TAZ.

Western blot analyses (Fig. 3C and D) showed a significant YAP1 (*p* < 0.01) and TAZ (*p* < 0.05) silencing, respectively, of around 50%, compared to WRAP5:siNEG, which was in coherence with the results shown in Fig. 2A and B. The 50% silencing was also observed when one of the two loaded siRNA was replaced by the siNEG.

**Fig. 4:**
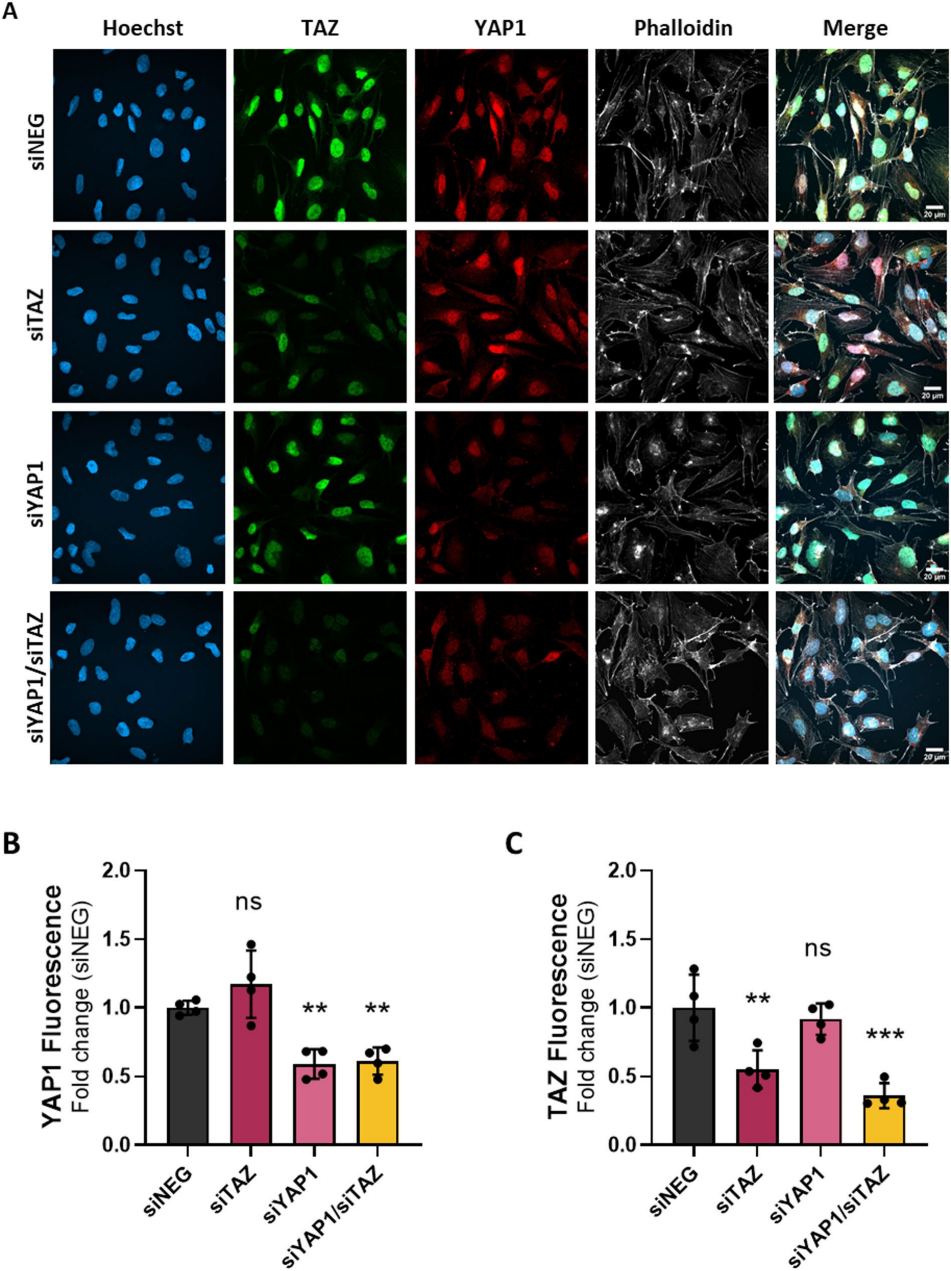
Immunofluorescence analysis of YAP1 and TAZ expression following the siRNA-mediated silencing in GIST-T1 cells. GIST-T1 cells were treated with WRAP5 nanoparticles loaded with 40 nM of siNEG (control), 20 nM of siYAP1 or siTAZ, or a combination of siYAP1 + siTAZ (20 nM each). Immunofluorescence staining was performed to detect endogenous YAP1 (red) and TAZ (green), with nuclei counterstained using Hoechst (blue) and Phalloidin (gray). Representative images are shown for each condition (**A**). Scale bars = 20 μm. Quantification of fluorescence intensity using ImageJ was performed for YAP1 (**B**) and TAZ (**C**) in the four conditions. Data were represented as mean ± SD for *N* = 2 with *n* = 2 each. Statistical analyses were performed using Kruskal-Wallis followed by Dunn’s multiple comparisons test *versus* siNEG.

To further confirm the specificity and efficiency of YAP1 and TAZ silencing at the cellular level, we performed immunofluorescence staining of both proteins in GIST-T1 cells treated with WRAP5 nanoparticles loaded with siNEG, siYAP1, siTAZ, or siYAP1 + siTAZ (Fig. 4A). Cells were co-stained for YAP1 and TAZ, and fluorescence intensity was quantified across individual cells. A significant decrease in TAZ signal (-45%, *p* < 0.01) was observed in cells treated with siTAZ (Fig. 4B), while YAP1 signal was significantly reduced (-40%, *p* < 0.01) in cells treated with siYAP1 (Fig. 4C) compared to the siNEG condition. Notably, no nuclear relocalization or compensatory accumulation of one protein was observed when the other was silenced. This absence of compensatory nuclear recruitment suggests that YAP1 and TAZ do not substitute for each other in GIST-T1 cells under these conditions. Finally, even if the fluorescence signal of TAZ (-64%, *p* < 0.0001) or of YAP1 (-40%, *p* < 0.01) seemed to be differently affected after (siYAP1 + siTAZ) treatment, the one-way ANOVA with multi-comparison revealed no significant difference *versus* siYAP1 or siTAZ conditions, respectively (Fig. 4B and C).

These results established WRAP5:siRNA nanoparticles as a reliable and non-toxic tool for selective and combinatorial gene silencing of YAP1 and TAZ in GIST cells, enabling us to dissect the role of both proteins individually or together in GIST biology.

### Selective effect of TAZ silencing on GIST-T1 cell migration and proliferation

After proving the effectiveness of our WRAP5:siRNA nanoparticles in silencing YAP1 and TAZ individually or together in GIST-T1 cells, we next investigated the functional consequences of their knockdown on cell migration and proliferation.

**Fig. 5:**
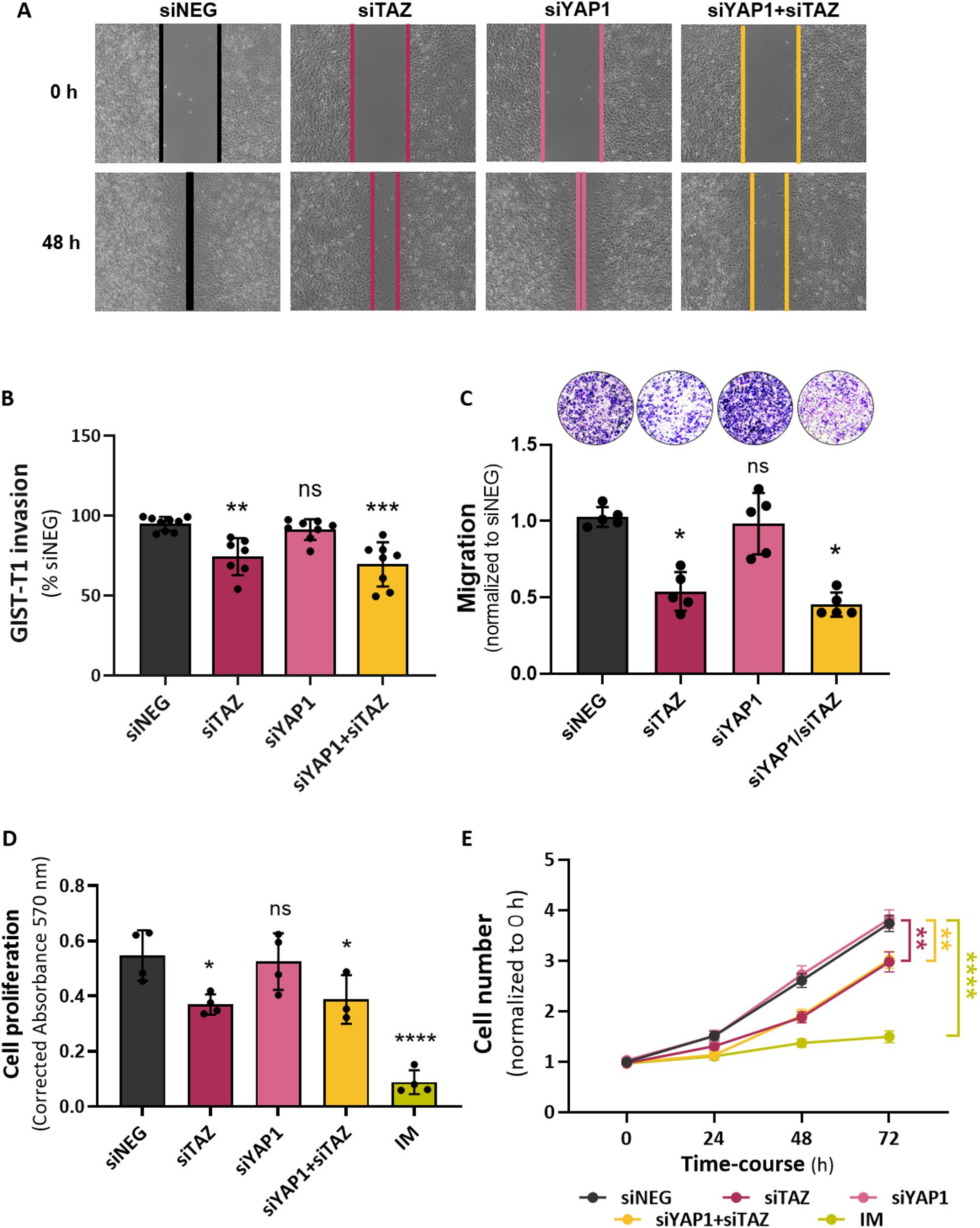
TAZ silencing reduced GIST-T1 cell migration and proliferation. (**A**) Representative images of the wound healing assay performed in GIST-T1 cells incubated with WRAP5:siRNA nanoparticles targeting TAZ, YAP1, or both (siYAP1+siTAZ). (**B**) The graph shows the percentage of wound closure relative to the initial scratch, normalized to the siNEG condition, as quantification of (A) (*N* = 8). (**C**) Effects of WRAP5:siRNA nanoparticles targeting TAZ, YAP1, or both (siYAP1+siTAZ) on migration were analyzed using the Transwell assay 36 h post-transfection in GIST-T1 cells (*N* = 5). Effects of WRAP5:siRNA nanoparticles targeting TAZ, YAP1, or both (siYAP1+siTAZ) on proliferation were analyzed using (**D**) the MTT assay, 72 h post-transfection (*N* = 4 with n=5 each) or (**E**) by cell counting over a 72 h time course period post-transfection (*N* = 5 with *n* = 2 each) in GIST-T1 cells.Conditions: WRAP5:siRNA (MR 20) with siRNA concentrations as indicated in the Materials & Methods section, siNEG was used as the control condition, IM (imatinib) = 200 nM. Data are presented as mean ± SD (**B**, **C**, **D**) or mean ± SEM (**E**). Statistical analyses were performed using Kruskal-Wallis followed by Dunn’s post-hoc test versus siNEG (B, C); one-way ANOVA followed by Holm-Sidak’s post-hoc test versus siNEG (**D**, **E**). ns >0.05, * <0.05, ** <0.01, *** <0.001, and **** <0.0001

To assess cell migration, we performed a wound healing assay on confluent GIST-T1 cells seeded on a culture insert to reproduce a homogeneous scratch (wound) (Fig. 5A). 48 h after “scratching” (= 72 h after siRAN transfection), we observed that TAZ silencing resulted in a substantial decrease (-24%) in wound closure compared to the siNEG control condition (*p* < 0.01), suggesting an impaired migratory capacity. With regard to YAP1 silencing, it is interesting to note that no significant effects (*p* = ns) have been observed, despite previous reports of its involvement in migration phenomena in other cancers [60, 61]. Co-silencing of YAP1 and TAZ using WRAP5:(siYAP1 + siTAZ) resulted in cell migration reduction (-31%) compared to the siNEG control (*p* < 0.001), which is equivalent to those observed for siTAZ (Fig. 5B). Results on cell migration were further evaluated using a Transwell assay performed 36 h after siRNA delivery to quantify the number of GIST cells migrating through an 8‑µm porous membrane toward a serum chemoattractant (Fig. 5C). GIST-T1 cells treated with WRAP5:siTAZ nanoparticles exhibited a significant decrease in the number of migrated cells compared with the siNEG control (-48%, *p* < 0.01), indicating that TAZ activity is required for efficient cell migration. In contrast, WRAP5:siYAP1 treatment did not alter migration. Combined silencing of YAP1 and TAZ produced a reduction similar to TAZ silencing alone, suggesting that the migratory defect is primarily driven by loss of TAZ. Together, these results confirmed observations of the wound healing assay showing that TAZ plays a predominant role in regulating GIST‑T1 cell migration.

We next evaluated cell proliferation, firstly using the MTT assay, an approach that measured metabolic cell activity with the same incubation conditions as used for the wound healing assay (Fig. 5D). Evaluation of the assay 48 h post-transfection showed no statistical differences between the WRAP5-based conditions (Figure S6). Only GIST-T1 cells incubated with IM (200 nM used as control) revealed a significant reduction in cell proliferation (-85%, *p* < 0.0001). In contrast, when the assay was evaluated 72 h post-transfection, a marked decline in proliferation rate was observed for TAZ silencing (-30%, *p* < 0.05) in comparison to the control condition siNEG or siYAP1. Interestingly, we observed an equivalent effect on proliferation when GIST-T1 cells were co-treated with (siYAP1 + siTAZ), also supporting the predominant role of TAZ (-20%, *p* < 0.05).

Similar results were observed when performing a direct cell counting over time (Fig. 5E). Following the transfection with WRAP5-based nanoparticles, YAP1 silencing alone did not result in a statistically significant reduction in cell proliferation. However, a reduction in the number of cells was observed 72 h post-transfection when TAZ was silenced alone or in combination with YAP1 (around − 21%, *p* < 0.01). No potential additive effect of the applied siYAP1 + siTAZ combination could be detected in both cell proliferation assays.

Together, these findings revealed that TAZ, more than YAP1, drives GIST-T1 cell proliferation and migration, and that its silencing had a stronger inhibitory effect on these tumorigenic properties.

### Impact of TAZ silencing on transcriptional target genes in GIST-T1 cells

Following the observed phenotypic effects of TAZ silencing on GIST-T1 cell migration and proliferation, we aimed to explore the molecular mechanisms underlying its function. Following the activation by phosphorylation, YAP1 and TAZ translocate into the nucleus, where they bind to the TEAD1-4 proteins, thereby facilitating the expression of downstream target genes (*CTGF*,* CYR61*,* AREG*,* MYC*,* MCL-1*,* BIRC5*,* AXL*) implicated in cell migration, proliferation, invasion, and resistance to apoptosis [23–25].

**Fig. 6:**
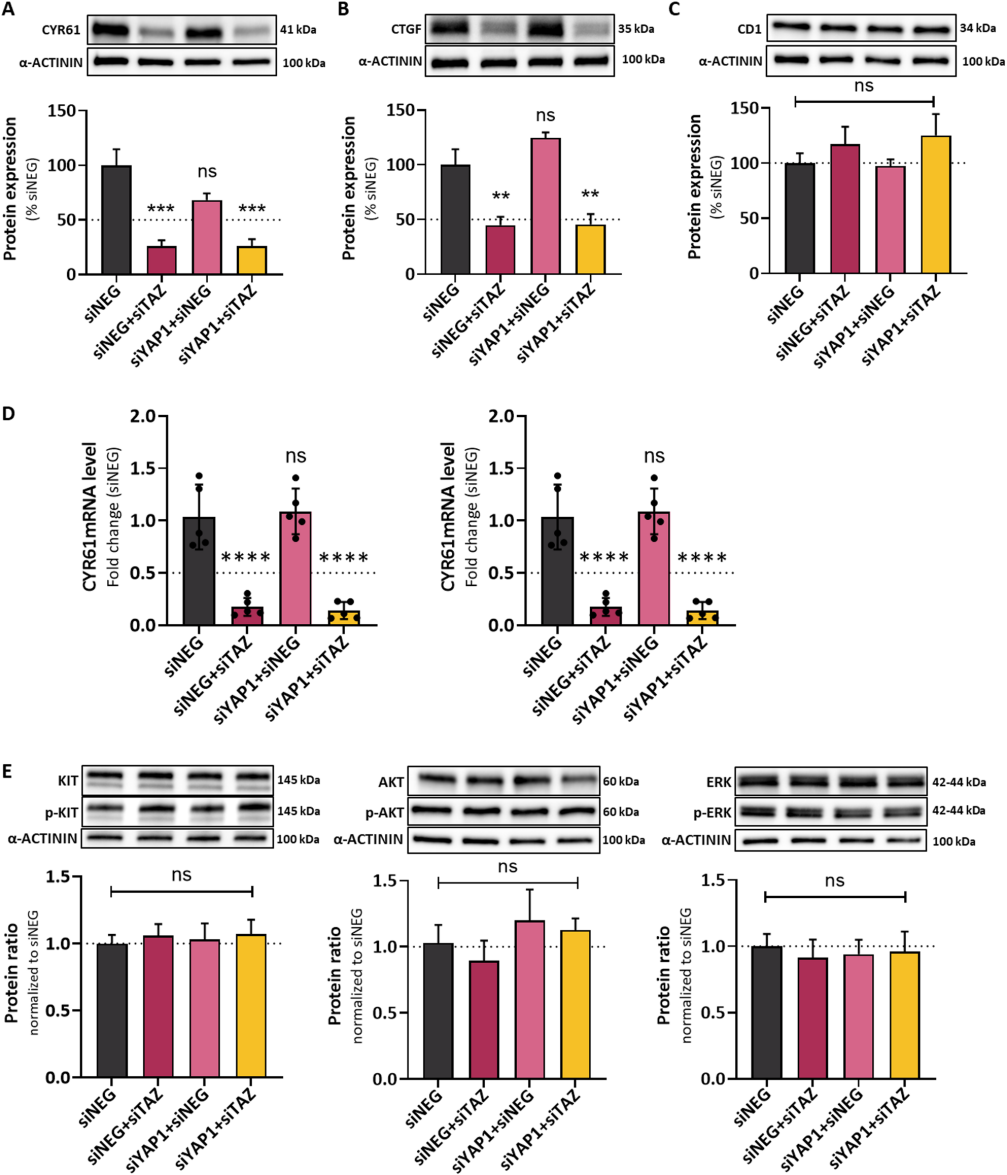
Evaluating TAZ silencing on different signaling pathways in GIST-T1 cells. Effect of WRAP5:siRNA nanoparticles targeting TAZ, YAP1, or both (siYAP1+siTAZ) on CYR61 (**A**), CTGF (**B**), and CD1 (**C**) protein expression in GIST-T1 cells was analyzed by Western blot analysis 48 h post-transfection. (**D**) Effect of WRAP5:siRNA nanoparticles targeting TAZ, YAP1, or both (siYAP1+siTAZ) on *CYR61* and *CTGF* mRNA levels in GIST-T1 cells was analyzed by qPCR analysis 24 h post-transfection. (**E**) Effect of WRAP5:siRNA nanoparticles targeting TAZ, YAP1, or both (siYAP1+siTAZ) on KIT signaling components (KIT, pKIT, AKT, pAKT, ERK, pERK) in GIST-T1 cells was analyzed by Western blot analysis 48 h post-transfection.Conditions: WRAP5:siRNA (MR 20 with [siRNA] = 40 nM), siNEG was used as the control condition. Data are presented as mean ± SD for *N *= 4-6. Statistical analyses were performed using one-way ANOVA followed by Dunnett’s post-hoc test versus siNEG, ns >0.05, ** <0.01, *** <0.001, ****<0.0001

As we observed an effect of TAZ on cell migration and proliferation, we first examined the expression of CYR61 and CTGF proteins. Western blot analyses on GIST-T1 cells incubated with the WRAP5 nanoparticles encapsulating the siYAP1 alone demonstrated no impact on CYR61 and CTGF expression (*p* = ns). Conversely, WRAP5:siTAZ nanoparticles revealed a significant downregulation of CYR61 protein (-65%, *p* < 0.001) and of CTGF (-55%, *p* < 0.01) compared to the control condition (WRAP5:siNEG) (Fig. 6A and B). This effect on CYR61 and CTGF protein expression was also observed when siTAZ was used in combination with siYAP1. All these results were confirmed by qPCR analyses that revealed a significant downregulation of *CYR61* and *CTGF* genes on mRNA levels upon TAZ knockdown, either alone or in combination with YAP1 (> 80%, *p* < 0.0001 for both, respectively) compared to WRAP5:siNEG. In contrast, YAP1 silencing alone had no measurable effect on *CYR61* mRNA level, but is associated with an increase of *CTGF* mRNA (Fig. 6D).

We also analyzed Cyclin D1 (CD1) expression to determine whether this key cell cycle regulator was modulated by YAP1 or TAZ in our model. Surprisingly, CD1 expression remained unchanged following the TAZ or YAP1 silencing (*p* = ns for all conditions *versus* siNEG), suggesting that its regulation in GIST-T1 cells was independent of these factors under the tested conditions (Fig. 6C).

Using a dual‑luciferase reporter assay, we evaluated if TEAD transcriptional activity was driven through TAZ silencing in GIST‑T1 cells. Indeed, we observed that WRAP5:siTAZ nanoparticles significantly decreased TEAD‑dependent luciferase signal compared with the siNEG control, demonstrating impaired TAZ–TEAD transcriptional output (Figure S7). The inhibitory effect of siTAZ was comparable to that of verteporfin, a known YAP/TAZ–TEAD disruptor, confirming the assay’s sensitivity.

Next, we evaluated the TAZ-CYR61 correlation in GIST-T1 cells by looking at WRAP5:siTAZ nanoparticles silencing when TAZ is overexpressed. After transient TAZ plasmid (pTAZ) transfection, treatment with siTAZ markedly reduced TAZ protein levels compared to the pNEG/siNEG control (Figure S8A). More importantly, TAZ overexpression was correlated with an overexpression of CYR61, which is again silenced after WRAP5‑mediated siTAZ delivery (Figure S8B). These results confirm that WRAP5:siTAZ nanoparticles not only overcome forced TAZ overexpression but also suppress its TEAD-transcriptional output.

Finally, to investigate the potential connection between the YAP1/TAZ and KIT signaling pathway, we assessed ratios of total and phosphorylated levels of KIT, AKT, and ERK, mimicking the activation of MAPK and PI3K/mTOR pathways. They represent the key effectors of KIT oncogenic signaling [24, 33]. No significant changes were observed following the YAP1 or TAZ knockdown in GIST-T1 cells (Fig. 6E), indicating that the anti-tumorigenic effects of TAZ silencing were independent of direct modulation of KIT or its canonical downstream cascades.

In conclusion, these results showed that TAZ promoted GIST-T1 proliferation and migration presumably through regulation of specific direct target genes, rather than by influencing CD1 and the KIT signaling axis. We thus hypothesized that CYR61 and CTGF proteins were specific downstream effectors of TAZ in GIST-T1 cells.

### Impact of YAP1/TAZ silencing in other GIST cells

**Fig. 7:**
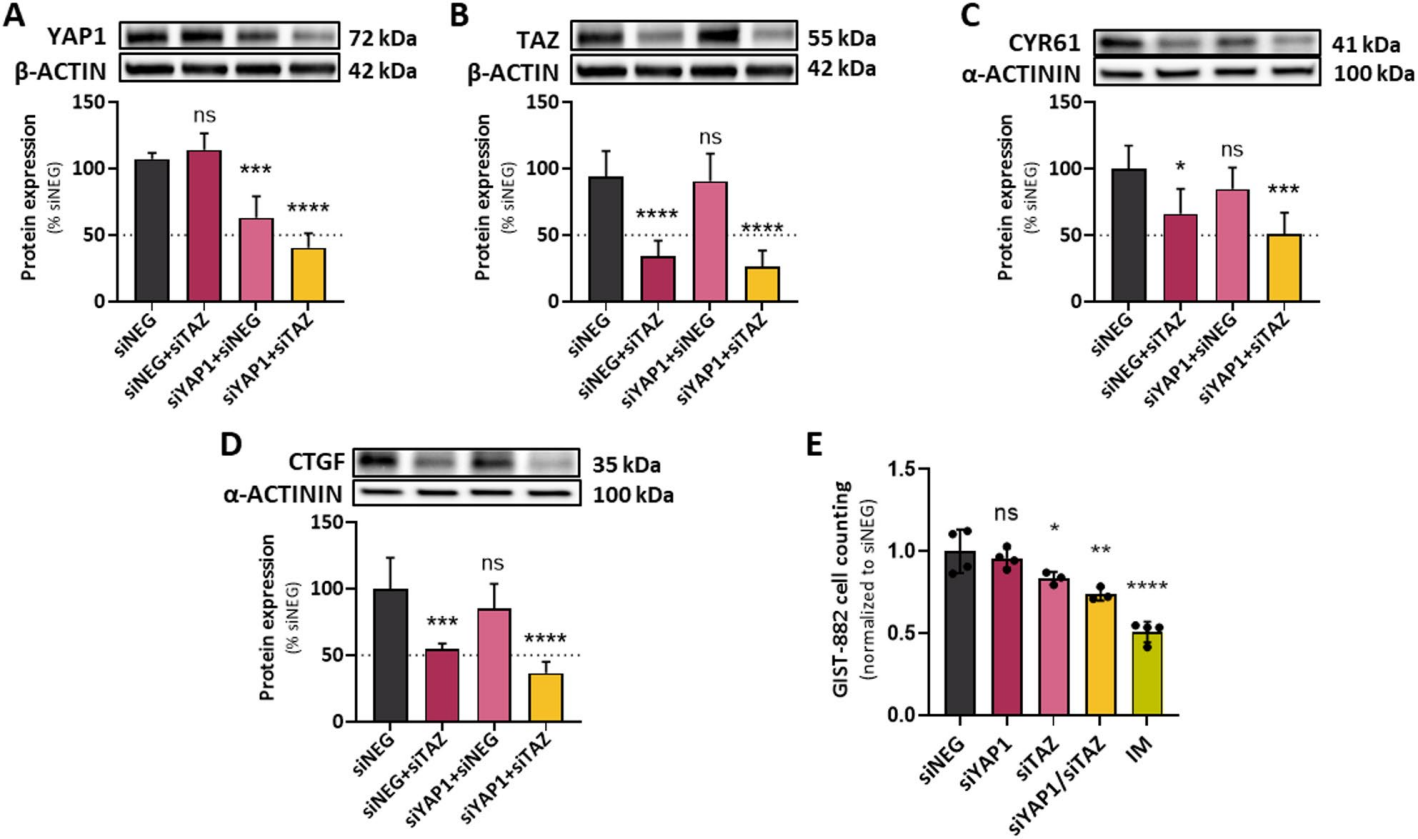
Effect of TAZ and YAP1 silencing in GIST-882 cells. Effect of WRAP5:siRNA nanoparticles targeting TAZ, YAP1, or both (siYAP1+siTAZ) on YAP1 (**A**) and TAZ (**B**), as well as on CYR61 (**C**) and CTGF (**D**) protein expression in GIST-882 cells was analyzed by Western blot 48 h post-transfection (*N* = 4-6). (**E**) Effects of WRAP5:siRNA nanoparticles targeting TAZ, YAP1, or both (siYAP1+siTAZ) on proliferation were analyzed by cell counting, 72 h post-transfection in GIST-882 cells (*N *= 4 with *n *= 2 each) in GIST-T1 cells. Conditions: WRAP5:siRNA (MR 20 with [siRNA] = 80 nM), siNEG was used as a control. Data are presented as mean ± SD. Statistical analyses were performed using one-way ANOVA followed by Dunnett’s post-hoc test versus siNEG (**A**, **B**, **C**, **D**), and one-way ANOVA followed by Holm-Sidak’s post-hoc test versus siNEG (**E**), ns >0.05, * <0.05, ** <0.01, *** <0.001, **** <0.0001

As the GIST pathology was characterized by different mutations, we decided to quantify YAP1 and TAZ protein level in GIST-430 and GIST-882 cells [51, 52]. These two cell lines harbor different *KIT* mutations than the primary mutation in the GIST-T1 cell (*KIT* exon 11 Δ560–578 in-frame deletion *versus* respectively KIT exon 11 Δ560–576 in-frame deletion and KIT exon 13 K642E mutation). These cells displayed distinct basal expression profiles of YAP1 and TAZ compared to GIST-T1 cells, as confirmed by Western blot analyses (Figure S9A). Notably, TAZ expression was markedly lower in GIST-882 cells, while both YAP1 and TAZ were expressed at higher levels in GIST-430, underscoring the molecular heterogeneity that characterized GIST tumors [62]. Importantly, total and phosphorylated KIT levels appeared comparable across the three cell lines, suggesting that differences in YAP1 and TAZ expression and downstream target regulation were not attributable to variations in KIT signaling (Figure S9B). However, given the differing morphologies (Figure S10) and proliferation rates of the three GIST cell lines, the protocol was adapted to each cell line in terms of cell density and siRNA concentrations (see Materials & Methods section for details). Using WRAP5:siRNA nanoparticles previously used, we evaluated YAP1 and TAZ silencing depending on the applied siRNA (siYAP1, siTAZ, siYAP1 + siTAZ, siNEG) in GIST-882 (Fig. 7A-B) and GIST-430 cells (Figures S12A-B). In both cell lines, YAP1 and TAZ knockdown were efficient when the corresponding siRNA was used compared to the siNEG (between 40% and 70%, depending on the condition used). We observed for the double knockdown, WRAP5:(siYAP1 + siTAZ) a slightly higher protein knockdown than with the single siRNA, which was not observed previously on GIST-T1 cells. However, when applying the one-way ANOVA statistical test followed by Dunnett’s post-hoc test with multiple comparisons, no significant difference was observed between these groups (data not shown).

Then, we also analyzed YAP1/TAZ silencing on downstream protein expression of CYR61 and CTGF in GIST-882 cells (Fig. 7C and D) and in GIST-430 (Figures S12C and S12D), as previously done for GIST-T1 cells. In detail, in GIST-882 cells, we observed a similar decrease for siTAZ and (siYAP1 + siTAZ), but here, we have no effect of YAP1 silencing on downstream CYR61 or CTGF expression (Fig. 7C and D). In GIST-430 cells, TAZ silencing as well as the double knockdown, WRAP5:(siYAP1 + siTAZ), significantly decreased both targets (between − 50% and 75% for CYR61 and for CTGF) (Figures S12C-D). YAP1 knockdown also affects these two downstream proteins, even to a lower manner (between − 30% and − 45%).

These results indicate that, although TAZ generally plays the predominant role in the three GIST cells, YAP1 may contribute more substantially in GIST‑430, where we observed a notably higher basal YAP1 expression (Figure S9A). This elevated expression suggests that YAP1 could assume a more prominent function in this particular cell line, acting in a complementary manner alongside TAZ.

Finally, we evaluated the effects of YAP1, TAZ, and combined YAP1 + TAZ silencing on the proliferation of GIST‑882 and GIST‑430 cells 72 h after treatment with WRAP5:siRNA nanoparticles. In GIST‑430 cells, YAP1 or TAZ silencing alone produced moderate reductions in proliferation (− 30%, *p* < 0.001 and − 36%, *p* < 0.001, respectively), whereas combined YAP1 + TAZ knockdown resulted in a more pronounced decrease (− 46%, *p* < 0.001), suggesting a complementary contribution of both proteins in this cell line (Fig. 7E). Proliferation analysis in the more slowly growing GIST‑882 cells revealed to be primarily dependent on TAZ, as TAZ silencing significantly reduced proliferation (− 17%, *p* < 0.05), while YAP1 knockdown had no detectable effect (Figure S12E). Dual silencing did not markedly exceed the impact of TAZ inhibition alone (− 26%, *p* < 0.01).

Together, these results indicate that the contributions of YAP1 and TAZ to GIST cell proliferation is cell‑line dependent, with GIST‑T1 and GIST‑882 cells showing predominant TAZ dependence, whereas GIST‑430 cell depending on both effectors.

### CYR61 as key downstream effector mediating TAZ-induced proliferation in GIST cells

**Fig. 8:**
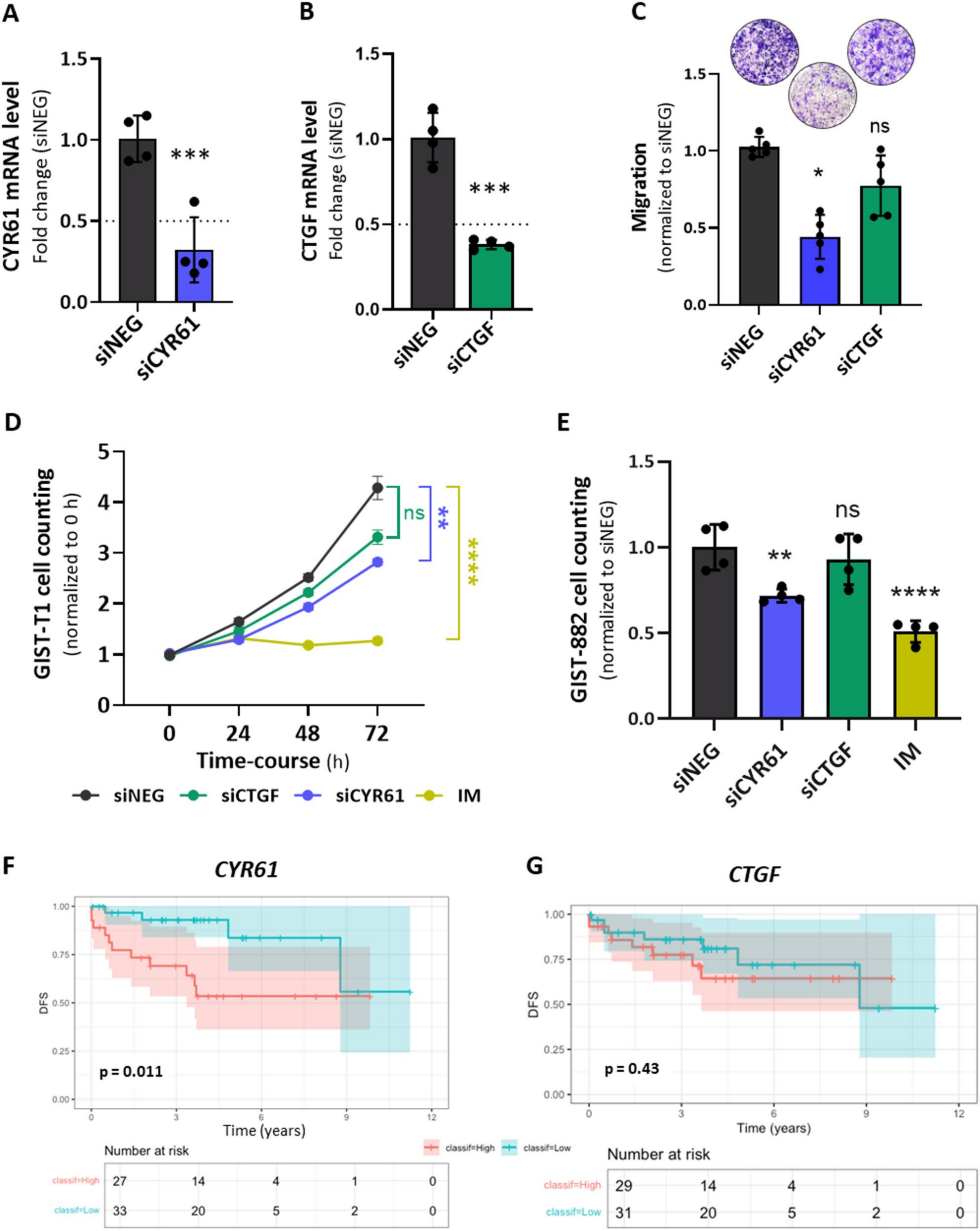
CYR61 is an effector of TAZ for GIST cell migration/proliferation and a marker of bad prognosis in GIST patients. The effect of WRAP5:siRNA nanoparticles targeting *CYR61* (**A**) and *CTGF* (**B**) mRNA levels in GIST-T1 cells was analyzed by qPCR analysis 24 h post-transfection (*N *= 6). **C** Effects of WRAP5:siRNA nanoparticles targeting CYR61 or CTGF on migration were analyzed using the Transwell assay 36 h post-transfection in GIST-T1 cells (*N *= 5). WRAP5:siRNA nanoparticles targeting CYR61 or CTGF were analyzed by cell counting in GIST-T1 cells over a 72 h time course post-transfection (**D**), in GIST-882 (**E**) cells at 72 h post-transfection (*N *= 4 with *n *= 2 each).Conditions: WRAP5:siRNA (MR 20) with [siRNA] = 20 nM for GIST-T1 and 80 nM for GIST-882 cells; siNEG was used as the control condition; IM (imatinib) = 50 nM. Data are presented as mean ± SD (**A**, **B**, **C**, **E**) or mean ± SEM (**D**). Statistical analyses were performed using unpaired two-tailed Student's t-test versus siNEG (**A**, **B**) using Kruskal-Wallis followed by Dunn’s multiple comparisons versus siNEG (**D**), or using one-way ANOVA followed by Dunnett’s post-hoc test versus siNEG (**E**); ns >0.05, ** <0.01, *** <0.001, **** <0.0001. (**F,G**) Kaplan-Meier curves of disease-free survival (DFS) from 60 total GIST patients in the ATGsarc microarray database, stratified by CYR61 or CTGF expression levels. Group 1 (red): patients with high gene expression (*CYR61n*=27 and *CTGFn*=29); Group 2 (blue): patients with low gene expression (*CYR61n*=33 and *CTGF n*=31). Number at risk = number of patients at risk over time. They are summarized in the table below the curves

We next silenced CYR61 and CTGF to ensure that they were effectors of the impact on cell proliferation observed for TAZ. For each gene, we designed two siRNA. Their specific knockdown efficiency and their potent cytotoxic effect were assessed by Western blot and by LDH assay, respectively (Figures S11). Two selected siRNA led to 90% and 60% of CYR61 and CTGF protein inhibition at the used concentration of 40 nM, respectively (Figures S11A-B and S11E-F) without any toxic effect on the cells (Figures S11C-D and S811G-H). The efficacy of these selected siRNA was also validated in qPCR analyses. This experiment led to significant downregulation of *CYR61* and *CTGF* mRNA levels 24 h after treatment with WRAP5:siCYR61 and WRAP5:siCTGF nanoparticles, respectively (-60%, *p* < 0.001 for both, Fig. 8A and B).

We next assessed the functional consequences of CYR61 or CTGF depletion on cell proliferation and migration in GIST‑T1 cells. In Transwell assays performed 36 h after nanoparticle exposure, CYR61 knockdown resulted in a significant and marked reduction in cellular migration (-57%, *p* < 0.001), whereas CTGF silencing produced only a modest and non‑significant effect (Fig. 8C). Cell‑counting analyses over a 72 h time course revealed that CYR61 suppression led to a substantial decrease in GIST‑T1 proliferation (− 34% at 72 h, *p* < 0.01), while CTGF silencing induced only a mild, non‑significant reduction in cell growth (around − 22% at 72 h, *p* = ns) (Fig. 8D). Importantly, these proliferative effects were validated in two other GIST cell lines, GIST‑882 (Fig. 8E) and GIST‑430 (Figure S13), in which CYR61 knockdown consistently impaired cell proliferation, further supporting its role as a conserved effector across genetically distinct GIST models. Finally, to explore the clinical relevance of these findings, we analyzed the same cohort of 60 patients from the ATGsarc microarray database [53] as previously performed for YAP1 and TAZ (Fig. 1). Kaplan-Meier survival analyses stratified patients by high versus low gene expression levels of *CYR61* or *CTGF*. High *CYR61* expression was significantly associated with reduced disease-free survival (*p* = 0.011; Fig. 8G) and with the presence of metastasis during GIST evolution (*p* = 0.0203; Figure S14A). In contrast, *CTGF* expression showed no significant correlation with either disease-free survival (*p* = 0.43) or metastatic status (*p* = 0.583) (Fig. 8H and S12B). *CYR61* is identified as a clinically relevant effector of TAZ, linking its overexpression to both increased tumor cell proliferation and poor clinical outcomes in GIST.

Our results indicate that CYR61 appears to function as an important downstream mediator of TAZ‑dependent proliferative effects in imatinib-sensitive GIST cells.

## Discussion

In this study, we provided the first investigation of the individual roles of YAP1 and TAZ, the two Hippo pathway effectors, in KIT-dependent IM-sensitive GIST cells. Indeed, it is well known that YAP1 and TAZ are implicated in cancer development and progression, even if most publications presented YAP1/TAZ as functionally redundant due to their structural similarity and shared transcriptional partners [24, 34, 35]. However, some reports suggest that both proteins have distinct roles, as reviewed by Reggiani et al. [37].

We aimed to evaluate whether it was possible to target YAP1 or TAZ separately using interfering RNA to elucidate which of these oncogenic proteins participated in GIST development and proliferation. Using our WRAP5-based nanoparticles as a delivery tool previously validated for therapeutic delivery [43–46], we vectorized specifically developed siRNA targeting the mRNA of *YAP1* and *TAZ*, individually, but also together using a siRNA cocktail containing an equimolar combination of both (siYAP1 + siTAZ).

With this tool, we observed a specific and significant silencing of YAP1 or TAZ individually or in combination in KIT-dependent IM-sensitive GIST-T1 (Fig. 3). However, only the silencing of TAZ resulted in a reduction in cell migration and cell proliferation (Fig. 5). More interestingly, transcriptional activation of downstream targets such as *CYR61* and *CTGF* genes was shown after TAZ silencing, while YAP1 exerted limited or nearly no effects on these genes implicated in cell proliferation. Notably, dual silencing of YAP1 and TAZ showed no additive effect on phenotypic outcomes in GIST-T1 cells. These results thus reinforce the idea of non-redundant roles in GIST cells and a more complex regulatory mechanism.

The functional divergence between YAP1 and TAZ protein function observed here was consistent with reports in other malignancies, especially cancers. In hepatocellular carcinoma and cholandiocarcinoma, only YAP1 played a crucial role in the development and progression [63, 64]. In non-small cell lung cancer, TAZ preferentially regulated genes involved in extracellular matrix remodeling and metastasis, whereas YAP1 controlled proliferation-related genes [65]. Moreover, the activation of TAZ nuclear translocation was associated with the highly aggressive triple-negative subtype of breast cancer [66]. Importantly, such divergence has also been described in sarcomas, a relevant context for GIST. For example, two research articles revealed an upregulated YAP1 expression level in osteosarcoma and contributed to cell differentiation [41, 67], even if the role of TAZ was not evaluated. In contrast, TAZ activity was modulated for the myogenic differentiation in rhabdomyosarcoma without the participation of YAP1 [68]. Our study extended this paradigm to GIST-T1 cells, establishing TAZ as a main oncogenic driver and supporting the notion of tissue- and lineage-specific YAP1/TAZ activity. However, our results supported the idea that TAZ could represent a more relevant therapeutic target in the context of GIST, in line with the clinical data identifying TAZ as a superior prognostic marker (Fig. 1).

Despite these findings, the mechanistic basis of the different functions between YAP1 and TAZ remained unclear, as both proteins mainly bound to TEAD transcription factors to regulate gene expression involved in cancer development. This raised the possibility that differential nuclear co-factor recruitment (co-activators or co-repressors) or divergent affinity for specific TEAD isoforms could underlie the selective transcriptional activity of TAZ [37, 61]. Further research should help to define these regulatory mechanisms, including chromatin context, transcriptional partner preferences, or post-translational modifications, which could fine-tune TEAD-dependent transcriptional output.

Across the three GIST cell lines analyzed, we observed marked heterogeneity in basal YAP1 and TAZ protein expression, which was reflected in their functional responses to silencing. Although TAZ consistently contributed to the repression of CYR61 and CTGF across models (Fig. 6A-B and D, and Fig. 7C-D, S12C-D), the extent of this effect varied depending on each cell line’s YAP1/TAZ expression balance. In GIST‑430, where YAP1 levels are relatively higher, the combined silencing of both effectors produced a more pronounced reduction in downstream gene expression and proliferation, suggesting that YAP1 can partially compensate for TAZ loss in this context. In contrast, GIST‑882 and GIST‑T1 exhibited a clearer dependence on TAZ, with YAP1 silencing alone exerting little measurable influence. Variations between GIST-882 and GIST-430 cells could also be attributed to their different responses to imatinib or to the HSP90 inhibitor, 17-allylamino-18-demethoxy-geldanamycin (17-AAG), as reported by Bauer and colleagues in 2006 [69].

This variability highlights the context‑dependent and potentially cooperative roles of YAP1 and TAZ in GIST biology. Such heterogeneity may mirror the molecular diversity observed in patient tumors and suggests that, in certain GIST subtypes, particularly those with elevated YAP1, dual targeting of YAP1 and TAZ could broaden pathway suppression. Similar context‑dependent benefits of dual inhibition have been reported in other cancers, where combined YAP1/TAZ blockade enhanced suppression of CYR61 expression and improved sensitivity to targeted therapies, including in KRAS G12C mutant models and in BRAF inhibitor‑resistant melanoma [70]. Likewise, in BRAF inhibitor-resistant melanoma, dual YAP1/TAZ silencing restored drug sensitivity and suppressed proliferation [71, 72].

While our data point toward circumstances in which combined YAP1/TAZ inhibition may be advantageous, we emphasize that potential synergy remains cell‑line specific. Further mechanistic and in *vivo* studies will be required to clarify when dual targeting provides meaningful therapeutic benefit in GIST.

In the present study, it was demonstrated that upon Hippo inactivation, the TAZ-CYR61 axis is activated, as evidenced by the proliferation assay (Fig. 8D-F). This activation exerts a negative impact on clinical progression, as shown by correlating TAZ/CYR61 expression in the GIST database for progression-free survival and metastasis formation (Fig. 8G). Indeed, the two genes are well-established downstream targets of the YAP1/TAZ-TEAD complex and are implicated in cell proliferation, angiogenesis, extracellular matrix deposition, and metastasis across multiple tumor types [24, 35].

In the three GIST cell models used, their expression was consistently regulated by TAZ and less by YAP1, reinforcing the specificity of TAZ-mediated transcription in GIST. A similar finding was revealed in gastric cancer, another digestive cancer, showing that TAZ upregulation was an important inducer of epithelial-mesenchymal transition and gastric cancer stem cells [56].

Although CD1 expression remained unaffected when YAP1 and TAZ were inhibited, this does not exclude the involvement of other cell-cycle regulators such as cyclin D2, cyclin D3, CDK2, or CDK4, notably in TAZ-driven proliferation [73]. Moreover, a modulation of apoptosis-related markers such as MCL-1 or BCL-2 could not be disregarded, leading to an indirect impact through other survival pathways [74]. Our results suggest that YAP1 and TAZ silencing does not significantly alter the major downstream signaling pathways of KIT activation, ERK/MAPK, and PI3K/AKT/mTOR, indicating that TAZ-driven transcriptional programs may promote tumor progression through pathways at least partly independent of canonical KIT signaling.

In our study, WRAP5:siRNA nanoparticles represent a promising experimental approach for targeted gene silencing in GIST. They exhibited low cytotoxicity and enabled efficient siRNA delivery, consistent with previous observations in GIST and other models [42, 44, 46–48, 75]. While these findings suggest that peptide‑based nanoparticles may expand the toolbox for RNA‑based modulation of YAP1/TAZ or their downstream transcriptional target genes, further comparative and in *vivo *studies will be required to fully assess their therapeutic potential. Nonetheless, this strategy may offer a complementary avenue to explore in a field where current treatments largely rely on tyrosine‑kinase inhibition [76]. Notably, siRNA-based drugs are now commonly accepted as six vectorized siRNA have been approved by the FDA (US Food and Drug Administration) and the EMA (European Medicines Agency) [77]. In the context of the presented siTAZ-loaded WRAP5-based nanoparticles as a therapeutic entity, further in *vivo *investigations will be needed to further promote their development, including in *vivo *delivery, pharmacokinetic evaluation, and therapeutic efficacy testing in appropriate GIST animal models.

Finally, to potentiate siRNA-loaded WRAP5 nanoparticles, especially for an in *vivo* application, other parameters should also be taken into account, such as the optimization of the nanoparticle itself through PEGylation (increasing the stability) or targeting motif insertion as reported previously in other physio-pathological contexts [46, 48, 75].

## Supplementary Information

Supplementary Material 1.

Supplementary Material 2.

Supplementary Material 3.

## Data Availability

GIST database (ATGsarc) is available at https://atg-sarc.sarcomabcb.org/.
