# Supplementary material for: Different contributions of YAP1 and TAZ in the regulation of GIST tumorigenic properties
Source: Cell Commun Signal. 2026 Apr 24;24:339. doi: 10.1186/s12964-026-02859-3 (PMC13245100; doi:10.1186/s12964-026-02859-3)
Supplement: Supplementary file 1 — Supplementary Material 1. [file 12964_2026_2859_MOESM1_ESM.zip › supp_file_raw_figures_R1.pptx]

## Slide 1
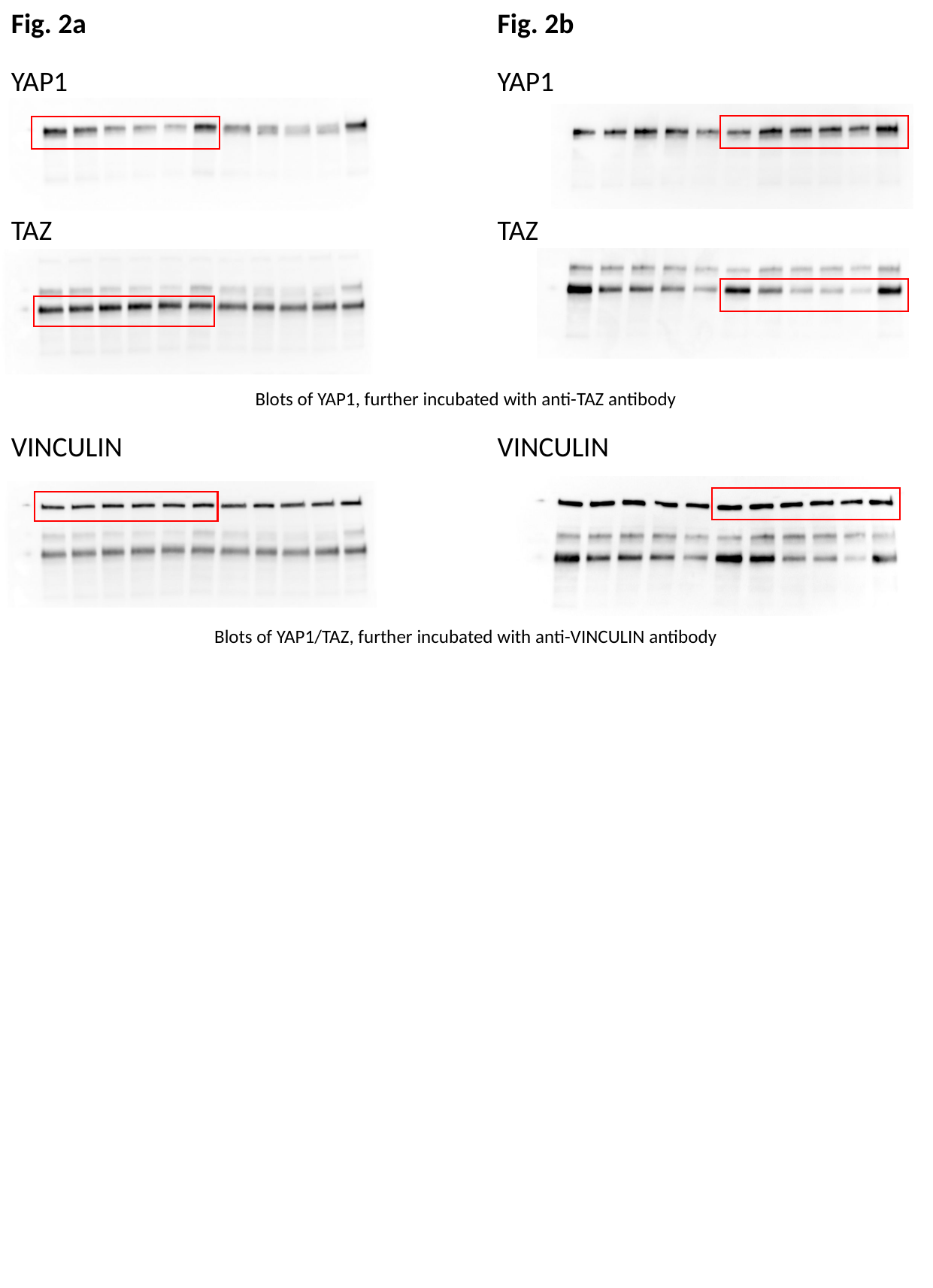

Fig. 2a
Fig. 2b
YAP1
YAP1
TAZ
TAZ
Blots of YAP1, further incubated with anti-TAZ antibody
VINCULIN
VINCULIN
Blots of YAP1/TAZ, further incubated with anti-VINCULIN antibody

## Slide 2
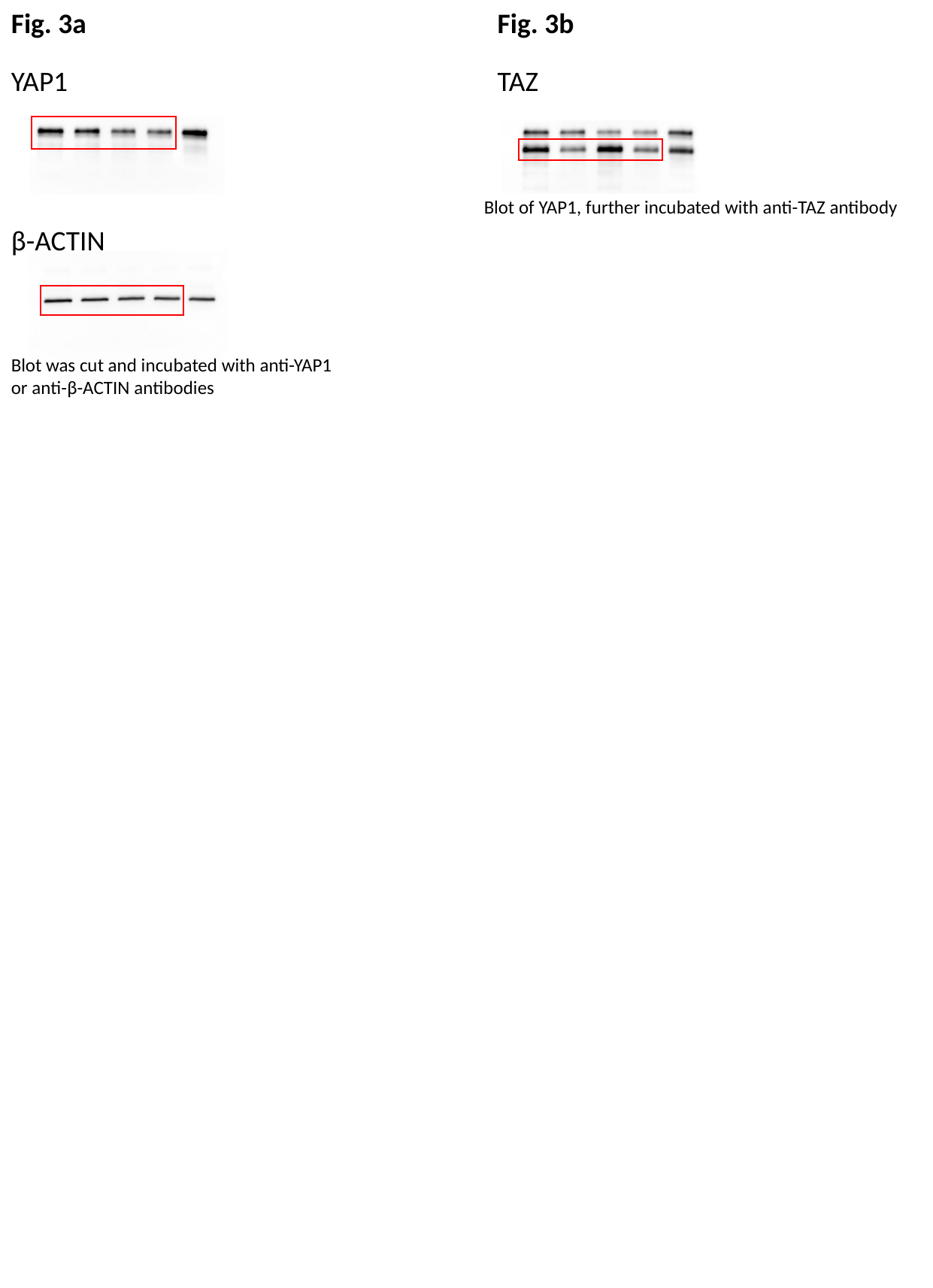

Fig. 3a
Fig. 3b
YAP1
TAZ
Blot of YAP1, further incubated with anti-TAZ antibody
β-ACTIN
Blot was cut and incubated with anti-YAP1 or anti-β-ACTIN antibodies

## Slide 3
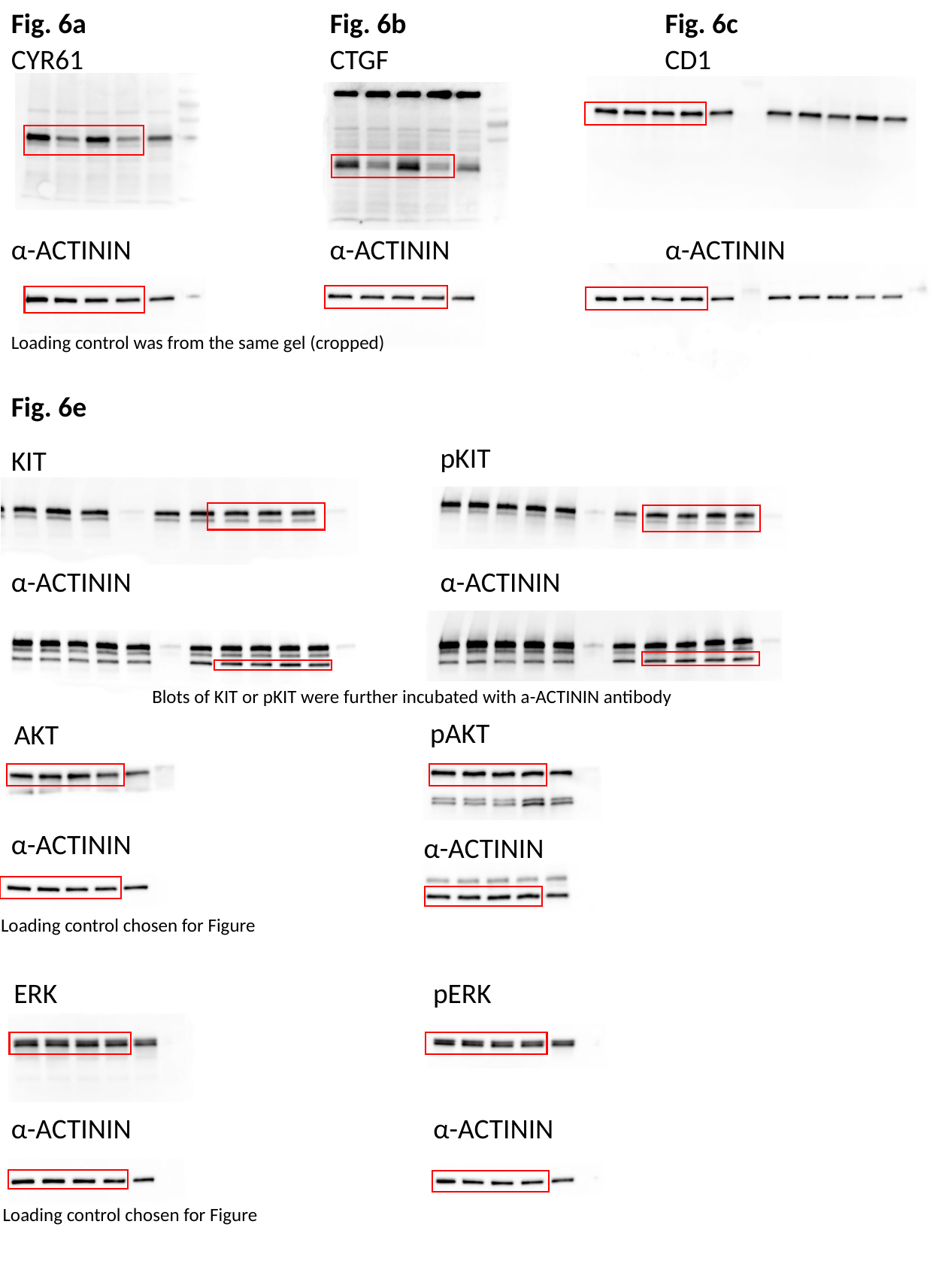

Fig. 6a
Fig. 6b
Fig. 6c
CYR61
CTGF
CD1
α-ACTININ
α-ACTININ
α-ACTININ
Loading control was from the same gel (cropped)
Fig. 6e
pKIT
KIT
α-ACTININ
α-ACTININ
Blots of KIT or pKIT were further incubated with a-ACTININ antibody
pAKT
AKT
α-ACTININ
α-ACTININ
Loading control chosen for Figure
ERK
pERK
α-ACTININ
α-ACTININ
Loading control chosen for Figure

## Slide 4
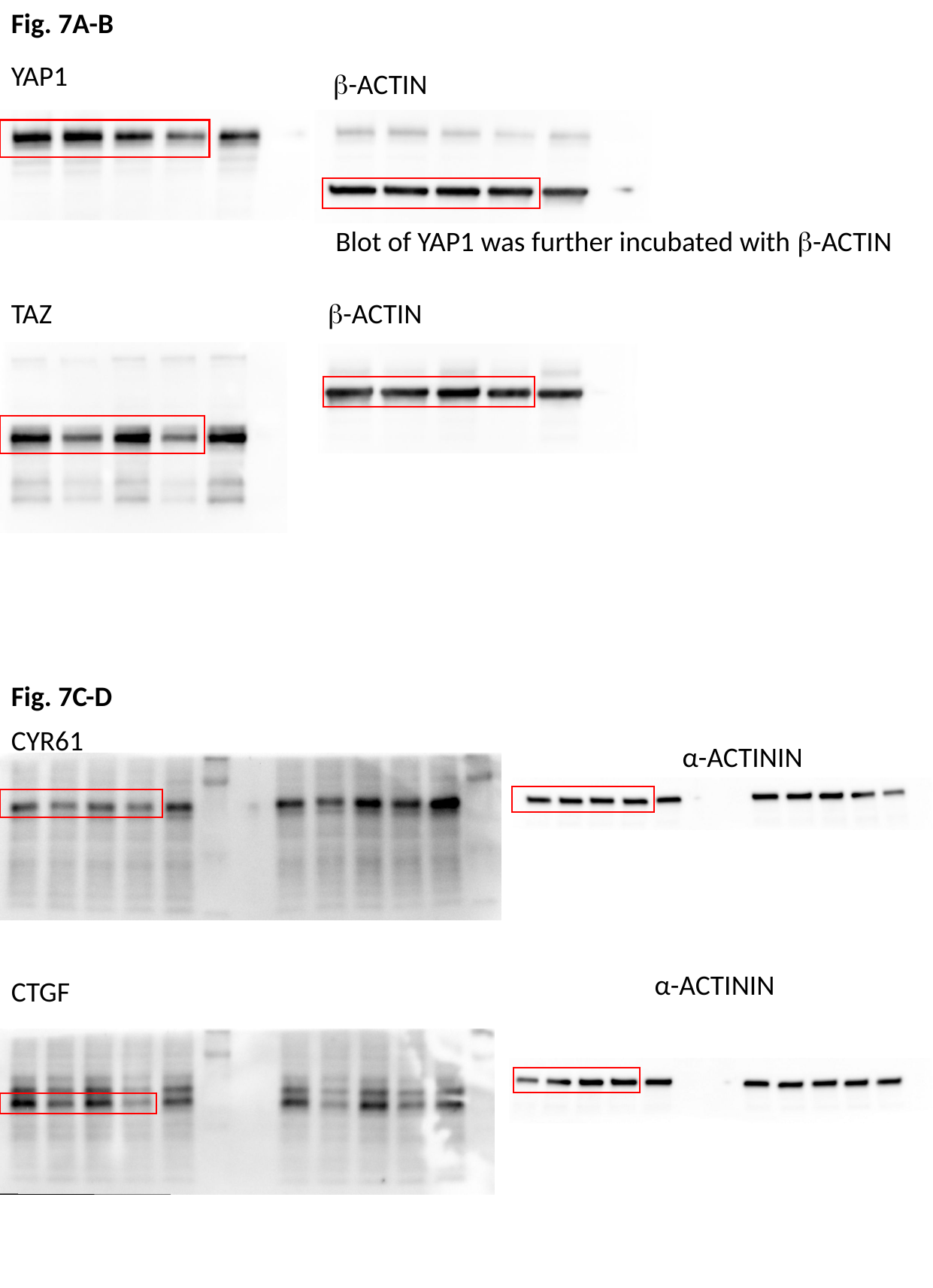

Fig. 7A-B
YAP1
b-ACTIN
Blot of YAP1 was further incubated with b-ACTIN
TAZ
b-ACTIN
Fig. 7C-D
CYR61
α-ACTININ
α-ACTININ
CTGF
