# Supplementary material for: Different contributions of YAP1 and TAZ in the regulation of GIST tumorigenic properties
Source: Cell Commun Signal. 2026 Apr 24;24:339. doi: 10.1186/s12964-026-02859-3 (PMC13245100; doi:10.1186/s12964-026-02859-3)
Supplement: Supplementary file 2 — Supplementary Material 2. [file 12964_2026_2859_MOESM2_ESM.zip › supp_file_raw_suppfigures_R1.pptx]

## Slide 1
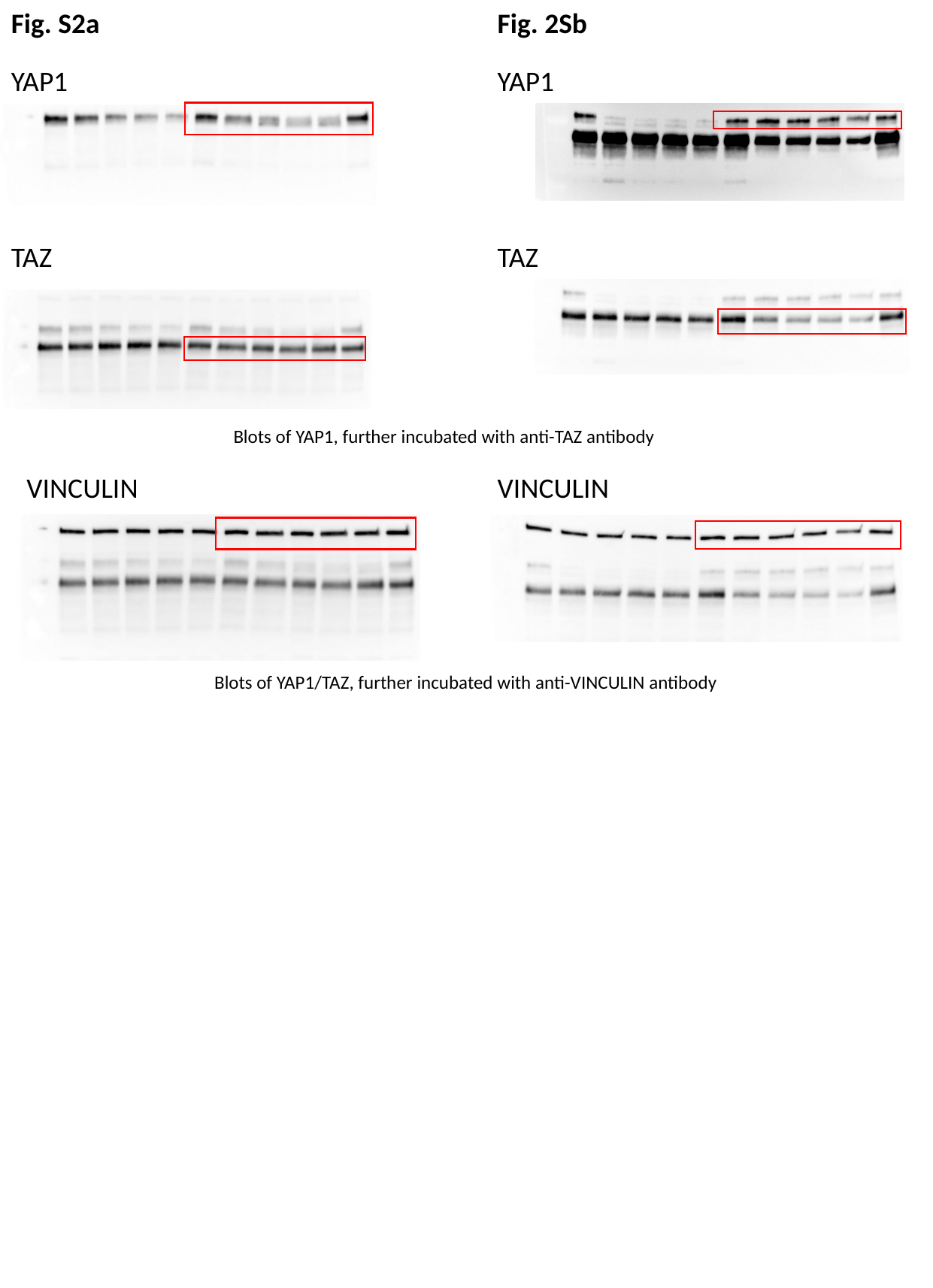

Fig. S2a
Fig. 2Sb
YAP1
YAP1
TAZ
TAZ
Blots of YAP1, further incubated with anti-TAZ antibody
VINCULIN
VINCULIN
Blots of YAP1/TAZ, further incubated with anti-VINCULIN antibody

## Slide 2
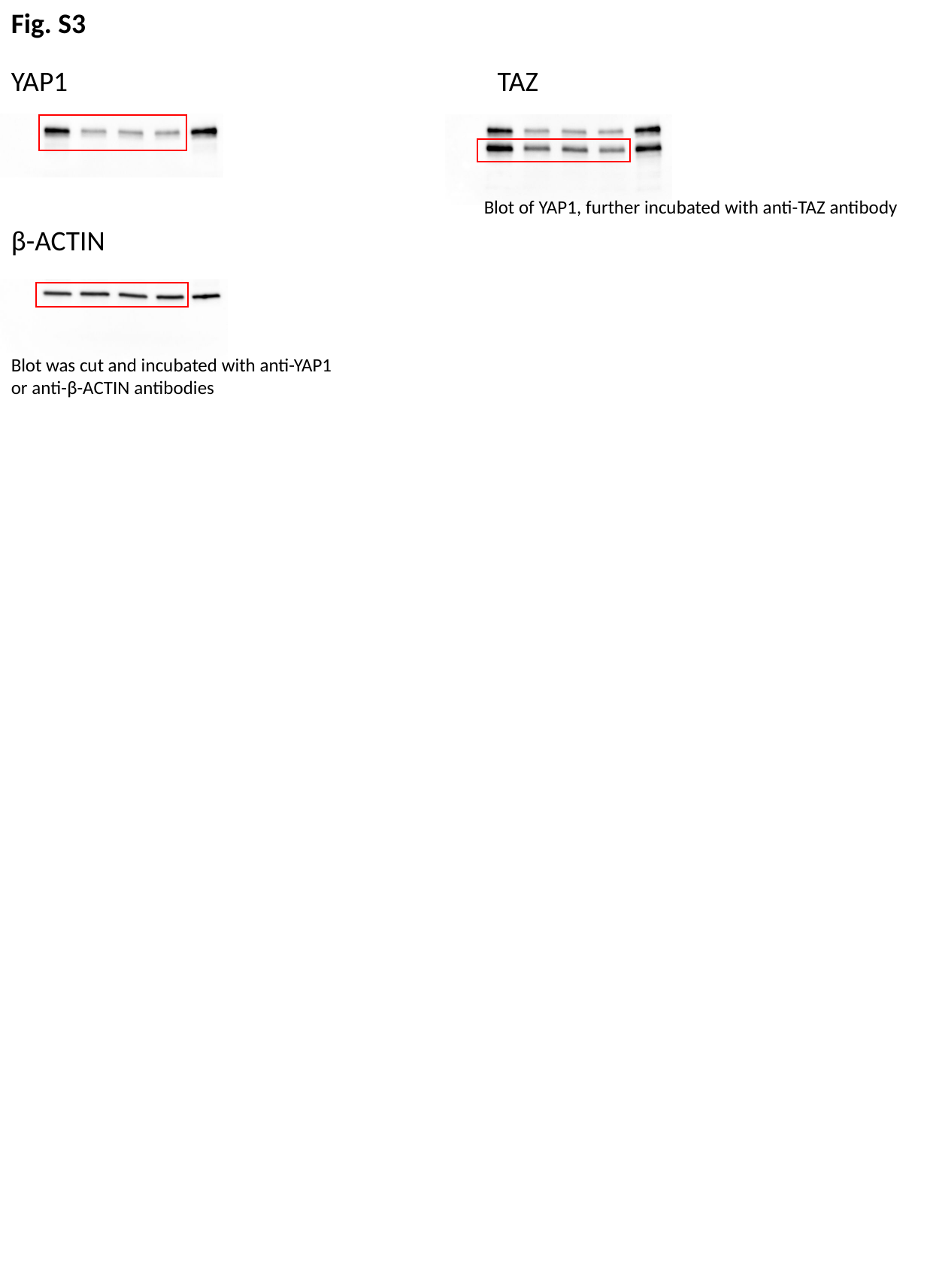

Fig. S3
YAP1
TAZ
Blot of YAP1, further incubated with anti-TAZ antibody
β-ACTIN
Blot was cut and incubated with anti-YAP1 or anti-β-ACTIN antibodies

## Slide 3
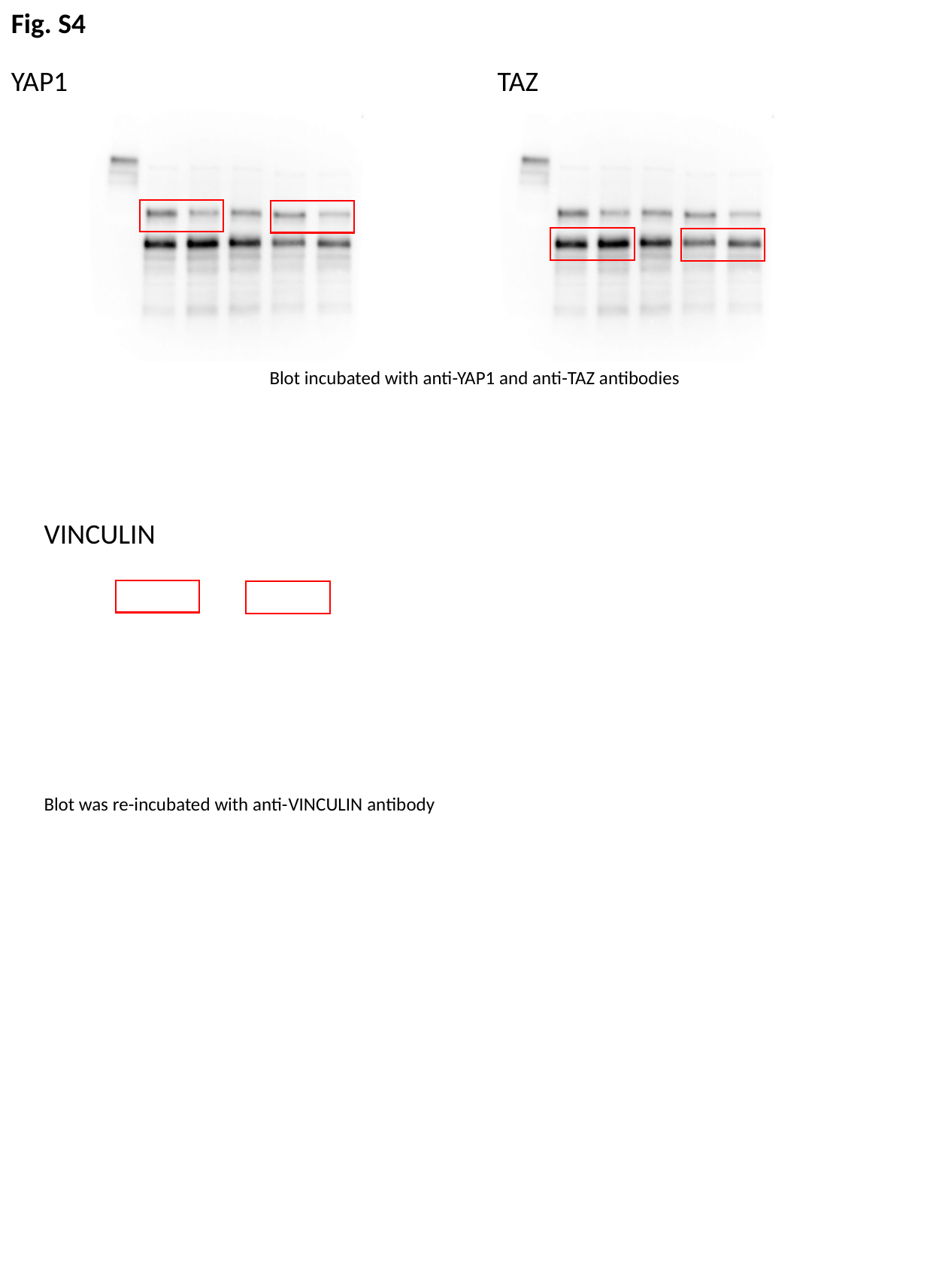

Fig. S4
YAP1
TAZ
Blot incubated with anti-YAP1 and anti-TAZ antibodies
VINCULIN
Blot was re-incubated with anti-VINCULIN antibody

## Slide 4
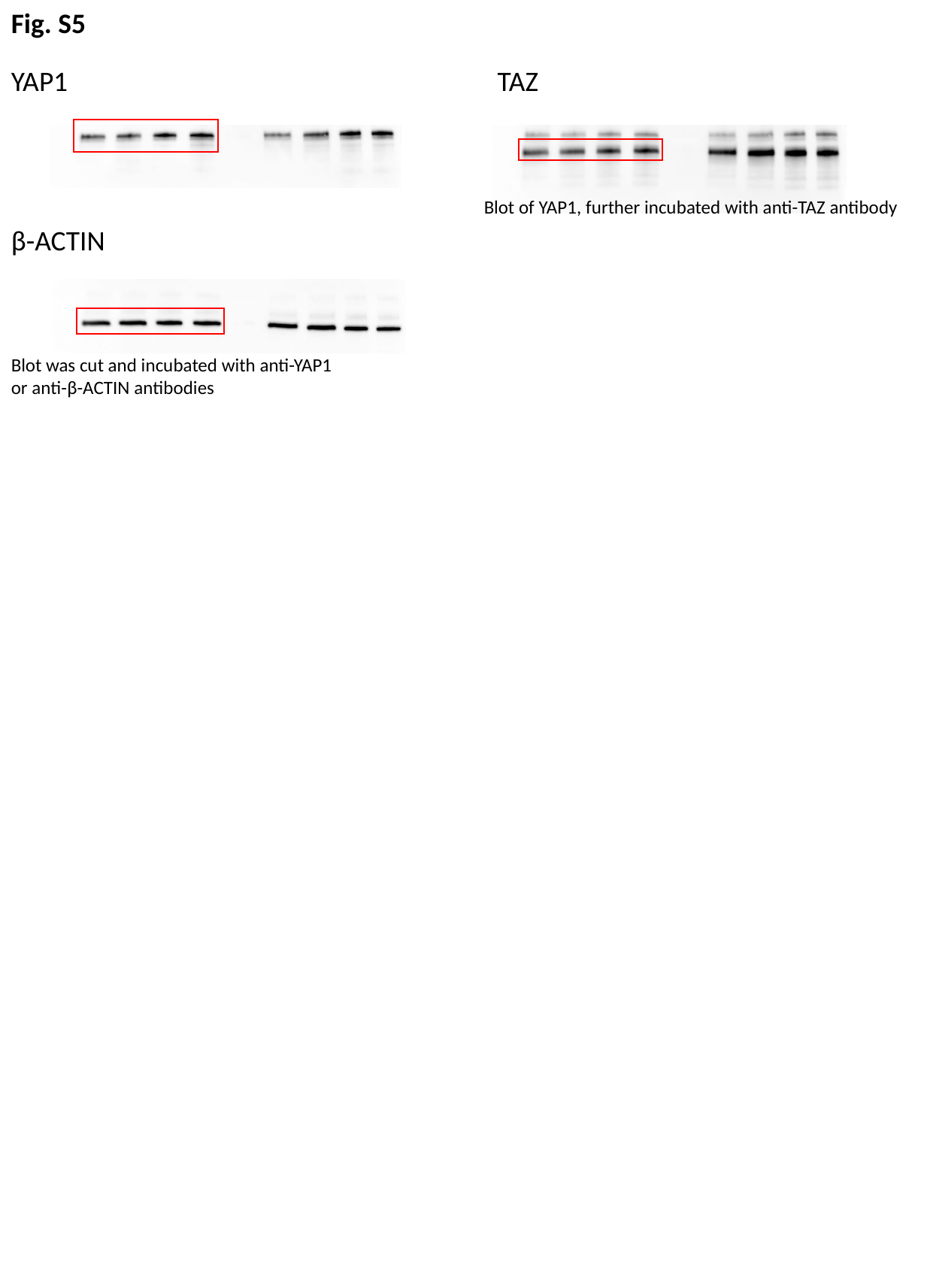

Fig. S5
YAP1
TAZ
Blot of YAP1, further incubated with anti-TAZ antibody
β-ACTIN
Blot was cut and incubated with anti-YAP1 or anti-β-ACTIN antibodies

## Slide 5
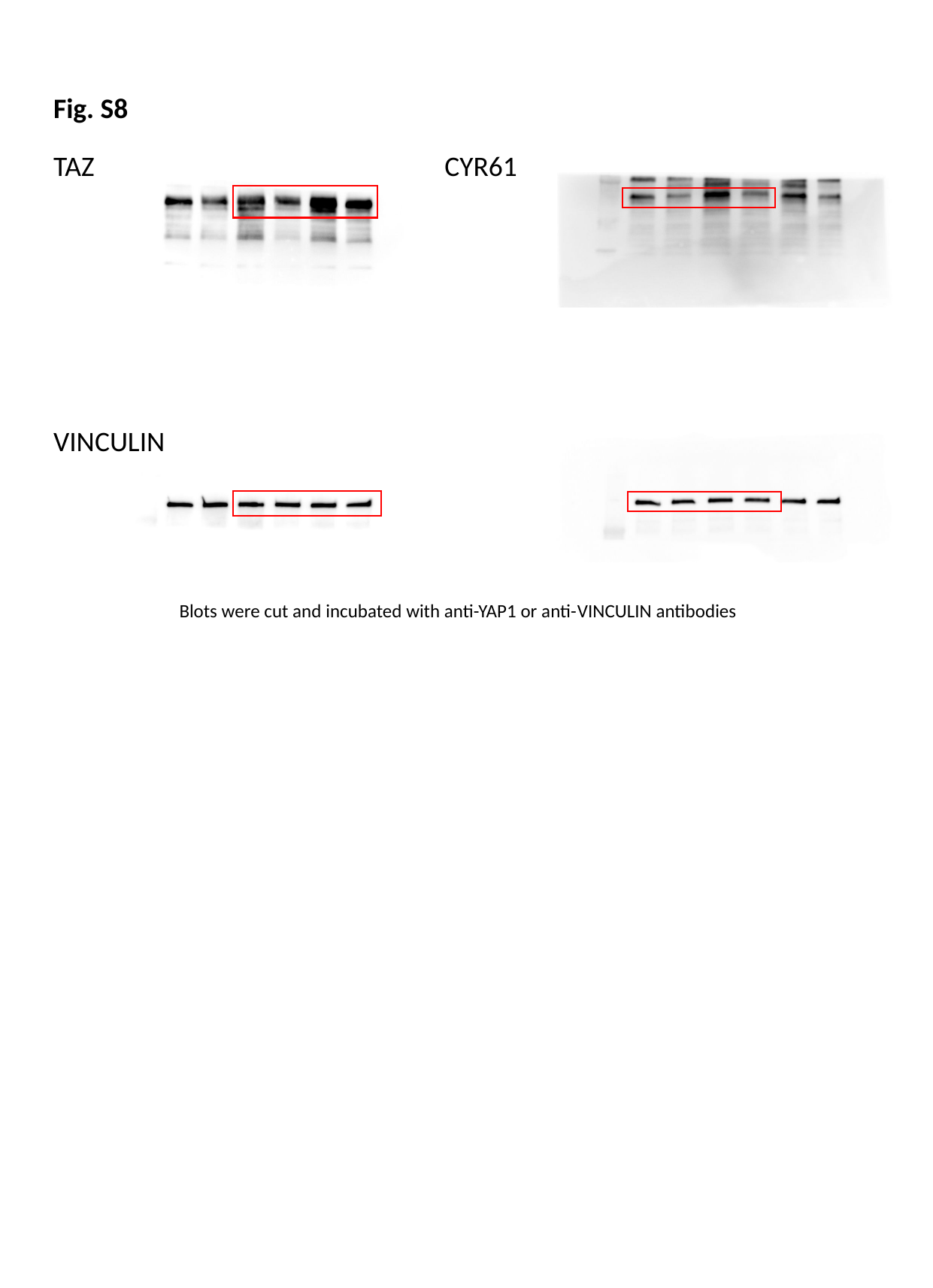

Fig. S8
TAZ
CYR61
VINCULIN
Blots were cut and incubated with anti-YAP1 or anti-VINCULIN antibodies

## Slide 6
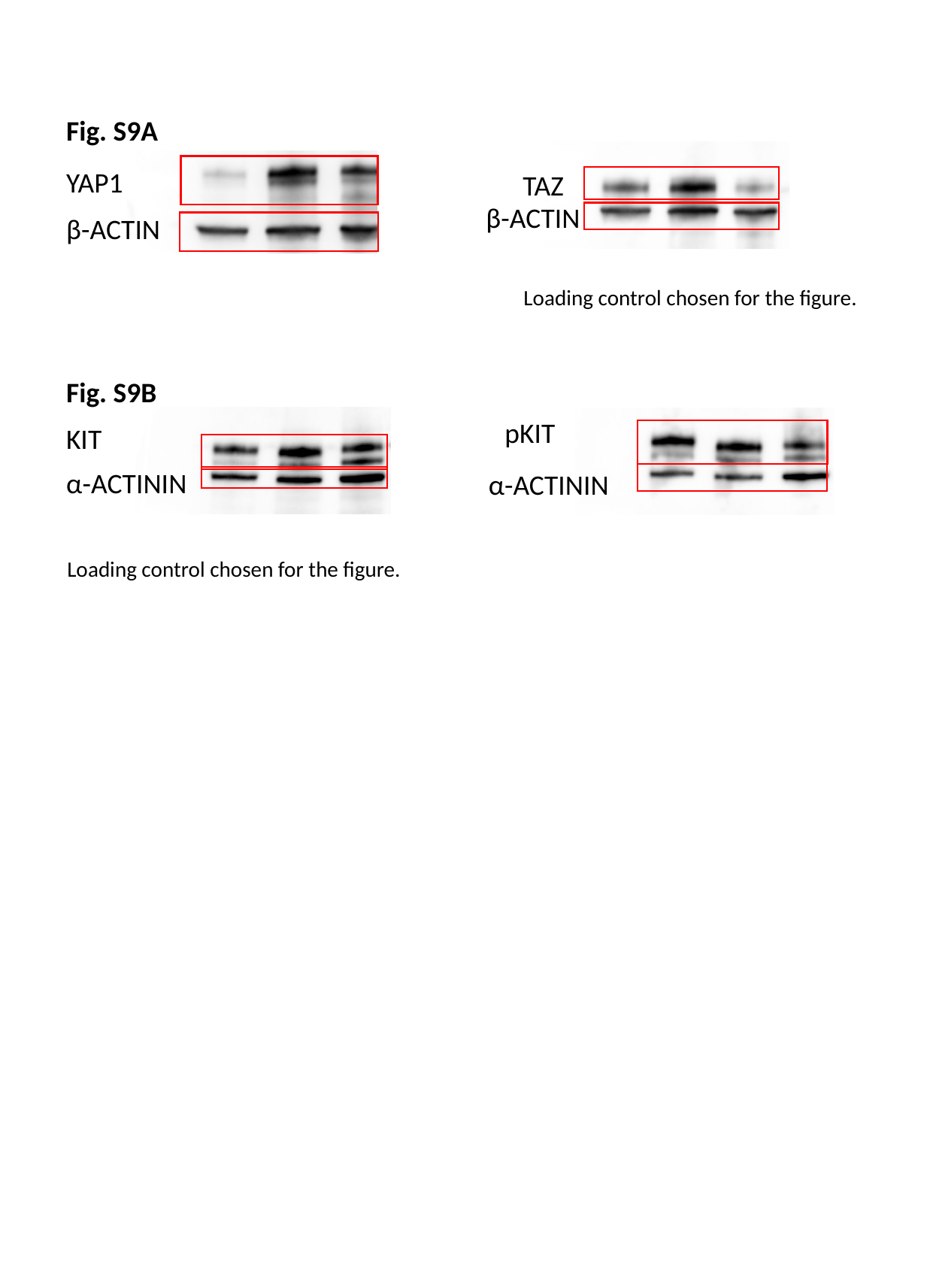

Fig. S9A
YAP1
TAZ
β-ACTIN
β-ACTIN
Loading control chosen for the figure.
Fig. S9B
pKIT
KIT
α-ACTININ
α-ACTININ
Loading control chosen for the figure.

## Slide 7
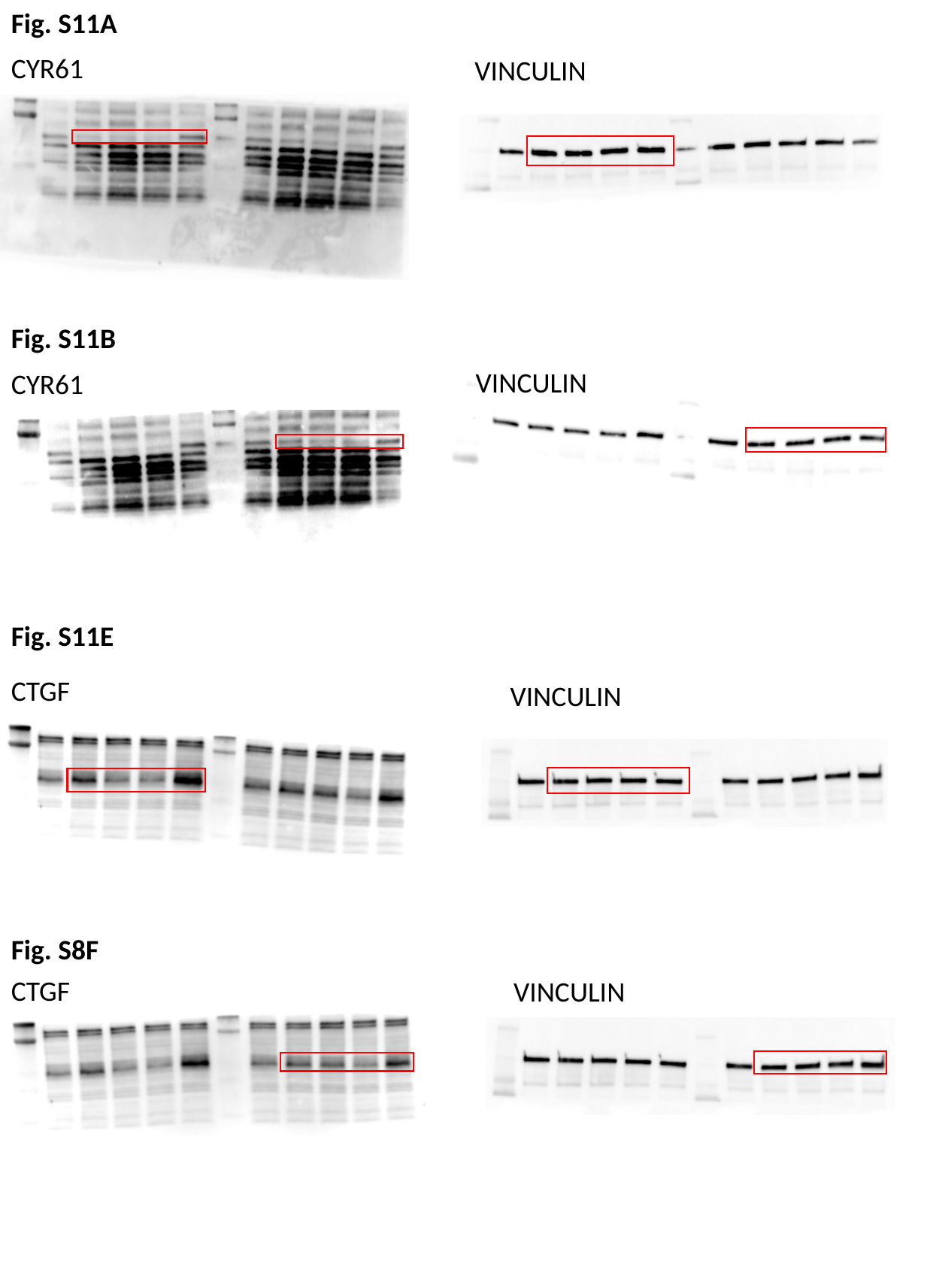

Fig. S11A
CYR61
VINCULIN
Fig. S11B
VINCULIN
CYR61
Fig. S11E
CTGF
VINCULIN
Fig. S8F
CTGF
VINCULIN

## Slide 8
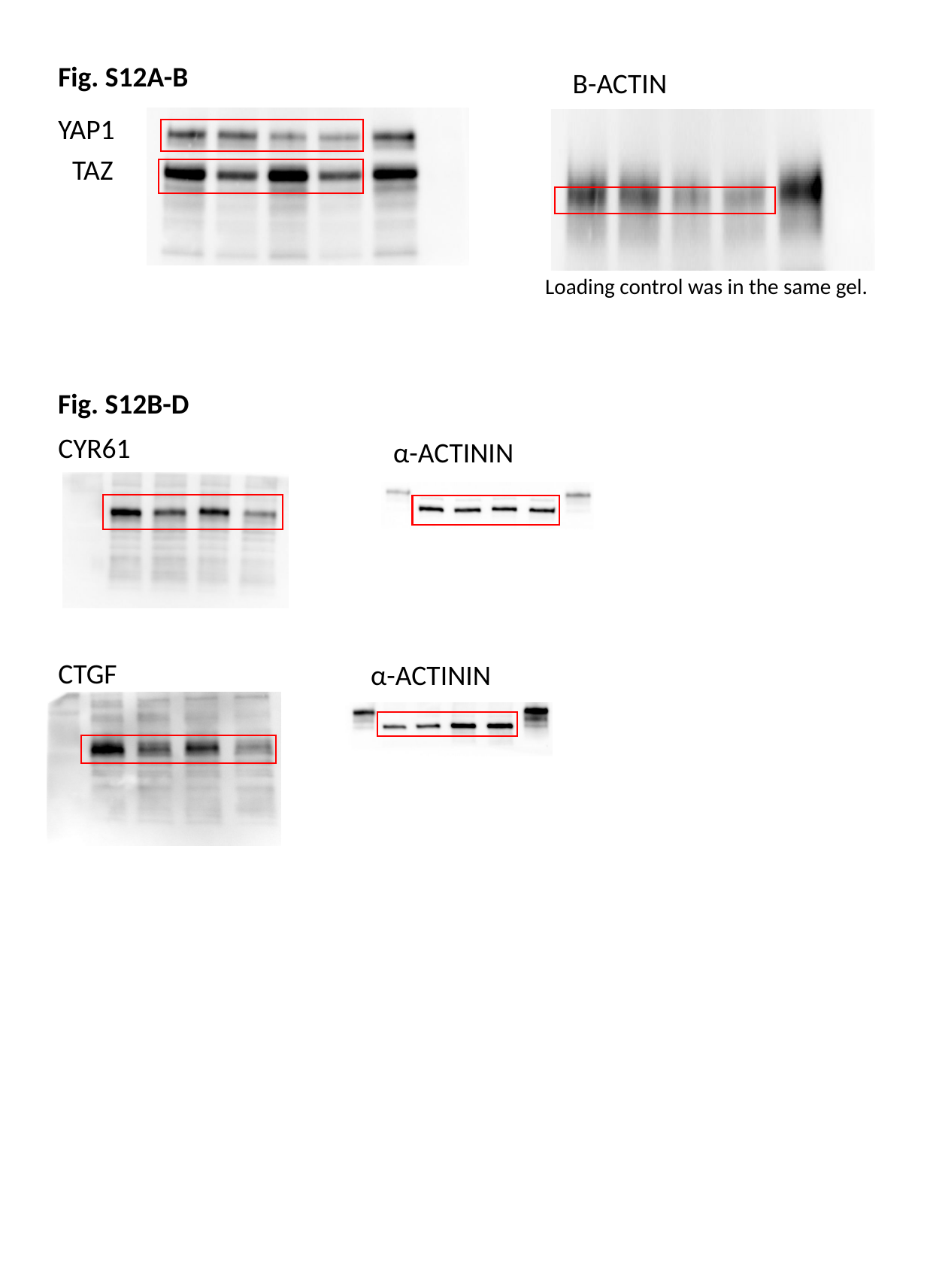

Fig. S12A-B
Β-ACTIN
YAP1
TAZ
Loading control was in the same gel.
Fig. S12B-D
CYR61
α-ACTININ
CTGF
α-ACTININ
