# Supplementary material for: Different contributions of YAP1 and TAZ in the regulation of GIST tumorigenic properties
Source: Cell Commun Signal. 2026 Apr 24;24:339. doi: 10.1186/s12964-026-02859-3 (PMC13245100; doi:10.1186/s12964-026-02859-3)
Supplement: Supplementary file 3 — Supplementary Material 3. [file 12964_2026_2859_MOESM3_ESM.pdf]

SUPPORTING INFORMATION

Supporting Methods

TAZ rescue experiment. For Western blot assays, 300,000 GIST-T1 cells were seeded into 6-well plates (Sarstedt, #83.3920) 24 hours before the experiment. For plasmid transfection, a solution of 100 µL, containing 0.75 µg p-TAZ (HA-TAZ, Addgene, #32839, kindly provided by A. Djiane, IRCM Montpellier) and JetPEI as transfection reagent (0.3 µL, PolyPlus, #POL101000053), was applied to GIST-T1 cells covered with 900 µL fresh pre-warmed complete medium. After a 6 h incubation period, the pDNA transfection solution was removed, and the cells were washed twice with DPBS. For nanoparticle incubation, cells were treated with 800 µL of fresh, pre-warmed serum-free culture medium and 200 µL of nanoparticle solutions at the specified concentrations, or 200 µL of 5% glucose when required. After 1.5 h, 1,000 µL of the culture medium supplemented with 20% FBS for GIST-T1 (final FBS concentration = 10%) was added to each well, without removing the transfection reagents. The cells were incubated for an additional 48 hours before being lysed for Western blot analysis.

TEAD-Luciferase reporter assay: TEAD transcriptional activity was evaluated using a dual-luciferase reporter assay as reported previously [1] with some modifications. 20,000 GIST-T1 cells were seeded in a 96-well plate and co-transfected with a Renilla luciferase reporter plasmid (pRL-SV40, Promega) and either a TEAD-responsive firefly luciferase reporter (pTEAD-Luciferase) or a TATA box control firefly luciferase reporter (TA-Luciferase) kindly provided by Prof. C. Varon (Bordeaux University). Transfections were carried out using JetPEI (PolyPlus, #POL101000053) with 10 ng of Renilla plasmid and 100 ng of either pTEAD-Luciferase or pTA-Luciferase per well (20 µL transfection solution in 80 µL complete medium). After 6 h, the JetPEI transfection media were discarded, and the cells were washed once with serum-free DMEM. Then, cells were incubated with 80 µL of serum-free DMEM and 20 µL of nanoparticle solutions. After 1.5 h, 100 µL of 20% serum-containing DMEM was added, without removing the transfection reagents. 48 h after transfection, cells were lysed, and luciferase activities were measured using the Dual-Luciferase Reporter Assay System according to the manufacturer's instructions (Promega, #E1960). Firefly luciferase activity was normalized to Renilla luciferase activity to control for transfection efficiency, and TEAD-specific transcriptional activity was calculated as the ratio of normalized TEAD activity to normalized TA activity.

Supporting Tables

Table S1: Used siRNA sequences

ID	Sense sequence	Anti-sense sequence	Reference
**siYAP1**	5’-CUA-UGU-UCA-UUC-CAU-CUC-CdTdT-3’	5’-GGA-GAU-GGA-AUG-AAC-AUA-GdTdT-3’	This work
**siYAP1’**	5’-AUA-GUA-AAU-UUC-UCC-AUC-CdTdT-3’	5’-GGA-UGG-AGA-AAU-UUA-CUA-UdTdT-3’	This work
**siTAZ**	5’-AAA-UCA-GGG-AAA-CGG-GUC-UdTdT-3’	5’-AGA-CCC-GUU-UCC-CUG-AUU-UdTdT-3’	This work
**siTAZ’**	5’-AUU-AUU-AGU-GAU-GGA-UCU-CdTdT-3’	5’-GAG-AUC-CAU-CAC-UAA-UAA-UdTdT-3’	This work
**si(YAPTAZ)**	5’-UGU-GGA-UGA-GAU-GGA-UAC-AdTdT-3’	5’-UGU-AUC-CAU-CUC-AUC-CAC-AdTdT-3’	(Tiffon et al., 2020)
**siCYR61**	5’-AUC-AUC-AUG-ACG-UUC-UUG-GdTdT-3’	5’-CCA-AGA-ACG-UCA-UGA-UGA-UdTdT-3’	This work
**siCYR61’**	5’-UGG-AGU-UGA-CGA-GAA-ACA-AdTdT-3’	5’-UUG-UUU-CUC-GUC-AAC-UCC-AdTdT-3’	This work
**siCTGF**	5'-AAU-UUA-GCU-CGG-UAU-GUC-UdTdT-3'	5'-AGA-CAU-ACC-GAG-CUA-AAU-UdTdT-3'	This work
**siCTGF’**	5’-AUU-UUG-GGA-GUA-CGG-AUG-CpTpT-3’	5’-GCA-UCC-GUA-CUC-CCA-AAA-UpTpT-3’	This work
**siNEG**	Confidential	Confidential	Negative Control siRNA, Eurogentec, #SR-CL000-005

Table S2: List of antibodies

Target (Protein)	MW (kDa)	Host Species	Clone / Catalog Number	Dilution	Company / Supplier	Application	RRID
**YAP1**	65 - 78	Rabbit	D8H1X / #14074	1:1,000 (GIST-T1) 1:5,000 (GIST-882) 1:10,000 (GIST-430)	Cell Signaling	WB	AB_2650491
**YAP1**	65-70	Mouse	66900-1	1:800	Proteintech	If	AB_2882229
**TAZ**	55	Rabbit	E9J5A / #72804	1:1,000 (GIST-T1 and GIST-882) 1:5,000 (GIST-430) 1:500 (If)	Cell Signaling	WB, If	AB_2904134
**KIT (CD117)**	145	Rabbit	#DB062	1:1,000	DB Biotech	WB	AB_2315699
**Phospho-KIT (pKIT)**	145	Rabbit	Tyr703 D12E12 / #3073	1:1,000	Cell Signaling	WB	AB_1147635
**AKT**	60	Rabbit	#9272	1:1,000	Cell Signaling	WB	AB_329827
**Phospho-AKT (pAKT)**	60	Rabbit	Ser473 D9E / #4060	1:1,000	Cell Signaling	WB	AB_2315049
**p44/42 MAPK (Erk1/2)**	42, 44	Mouse	L34F12 / #4696	1:1,000	Cell Signaling	WB	AB_390780
**Phospho-p44/42 MAPK (Erk1/2)**	42, 44	Rabbit	Thr202/Tyr204 20G11 / #4376	1:1,000	Cell Signaling	WB	AB_331772
**CYR61**	41	Rabbit	D4H5D / #14479	1:1,000	Cell Signaling	WB	AB_2798492
**CTGF**	35	Rabbit	D8Z8U / #86641	1:1,000	Cell Signaling	WB	AB_2800085
**Cycline D1 (CD1)**	34	Rabbit	SP4 / #MA5-16356	1:1,000	ThermoFisher Scientific	WB	AB_2537875
**VINCULIN**	124	Rabbit	E1E9V / #13901	1:1,000	Cell Signaling	WB	AB_2728768
**α-ACTININ**	100	Rabbit	D6F6 / #6487	1:1,000	Cell Signaling	WB	AB_11179206
**β-ACTIN**	42	Mouse	#A5441	1:10,000	Sigma-Aldrich	WB	AB_476744
**Anti-Rabbit**	/	IgG, HRP	#7074	1:1,000	Cell Signaling	WB	AB_2099233

**Anti-Mouse**	/	IgG, HRP	#7076	1:10,000 (β-Actin) 1:1,000 (others)	Cell Signaling	WB	AB_330924
**Anti-Rabbit Alexa Fluor™ 488**	/	Donkey	#A31570	1:1,000	Invitrogen	If	/
**Anti-Mouse Alexa Fluor™ 555**	/	Donkey	#A21206	1:1,000	Invitrogen	If	/

Footnotes: WB: Western Blot / If: Immunofluorescence.

Table S3: Human gene-specific primers used for RT-qPCR.

Target	Forward primer (5'-3')	Reverse primer (5'-3')	Amplicon (bp)	Reference
**YAP1**	CACAGCATGTTCGAGCTCAT	GATGCTGAGCTGTGGGTGTA	120	(Molina-Castro et al., 2020)
**TAZ**	TGCCGTCAGTTCCACAC	GTTCTGCTGGCTCAGGGT	115	This work
**CYR61**	GGAGCCTCGCATCCTAT	ATTGGTAACTCGTGTGGAGA	115	(Guérin et al., 2020)
**CTGF**	TTCAAGTGCCCTGACGG	GCGATTCAAAGATGTCATTGTCTC	117	This work
**HPRT1**	TGGTCAGGCAGTATAATCCA	GGTCCTTTTCACCAGCAAGCT	59	(Molina-Castro et al., 2020)

REFERENCES

1. Giraud J, Molina-Castro S, Seeneevassen L, Sifré E, Izotte J, Tiffon C, et al. Verteporfin targeting YAP1/TAZ-TEAD transcriptional activity inhibits the tumorigenic properties of gastric cancer stem cells. Int J Cancer. 2020;146:2255–67. https://doi.org/10.1002/ijc.32667
2. Guérin, A., Martire, D., Trenquier, E., Lesluyes, T., Sagnol, S., et al. LIX1 regulates YAP activity and controls gastrointestinal cancer cell plasticity. Journal of Cellular and Molecular Medicine. 2020;24(16), 9244-9254. https://doi.org/10.1111/jcmm.15569
3. Molina-Castro, S. E., Tiffon, C., Giraud, J., Boeuf, H., Sifre, E., et al. The Hippo Kinase LATS2 Controls Helicobacter pylori-Induced Epithelial-Mesenchymal Transition and Intestinal Metaplasia in Gastric Mucosa. Cellular and Molecular Gastroenterology and Hepatology. 2020;9(2), 257-276. https://doi.org/10.1016/j.jcmgh.2019.10.007
4. Tiffon, C., Giraud, J., Molina-Castro, S. E., Peru, S., Seeneevassen, L., et al. (2020). TAZ Controls Helicobacter pylori-Induced Epithelial-Mesenchymal Transition and Cancer Stem Cell-Like Invasive and Tumorigenic Properties. Cells 2020;9(6), 1462. https://doi.org/10.3390/cells9061462

Supporting Figures

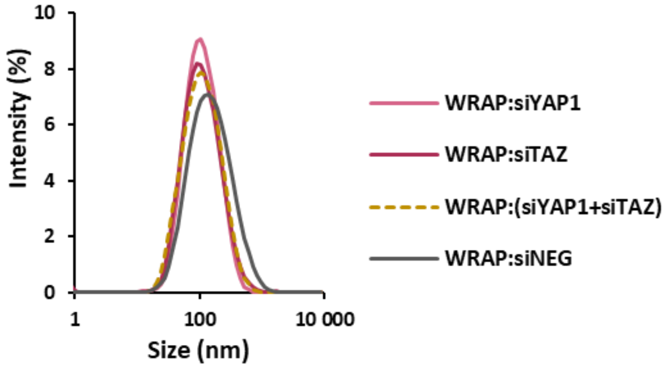

Size (nm)	PdI
92.7 ± 2.1	0.29 ± 0.06
92.8 ± 11.9	0.30 ± 0.04
89.3 ± 8.9	0.29 ± 0.02
121.9 ± 7.3	0.36 ± 0.01

Figure S1: Characterization of WRAP5:siRNA nanoparticles by dynamic light scattering (DLS).
Mean size distribution of WRAP5:siRNA nanoparticles (W5 = 10 µM, MR = 20)
DLS measurements were performed on all nanoparticles, with three individual experiments (N = 3) and three runs per condition.

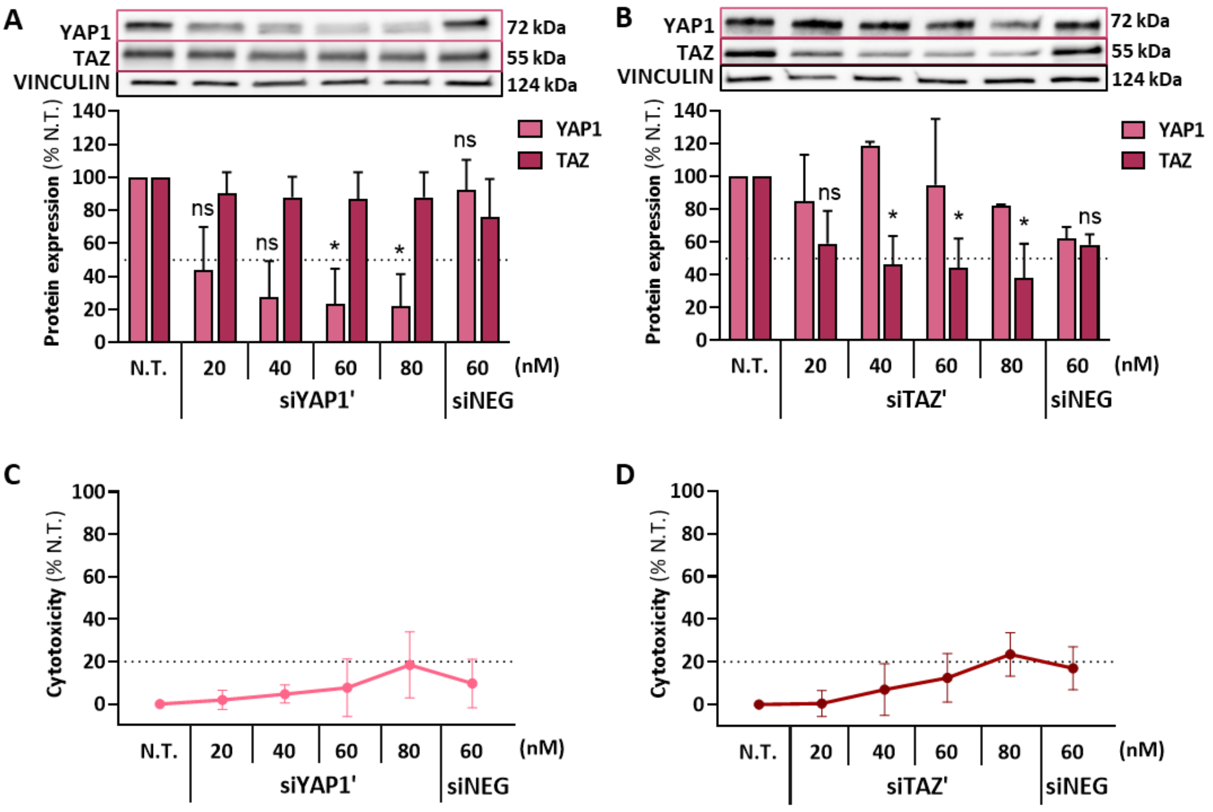

Figure S2: Evaluation of YAP1 or TAZ silencing in GIST-T1 cells with WRAP5:siRNA’ nanoparticles
WRAP5:siRNA nanoparticles delivering siYAP1’ (**A**) or siTAZ’ (**B**) induced a dose-dependent inhibition of YAP1 or TAZ in GIST-T1 cells after 48 h of incubation, as shown by Western blot quantification. A slight toxicity was observed respectively for siYAP1’ (**C**) and siTAZ’ (**D**) at 80 nM and above, as assessed by LDH assay.
Controls include untreated (N.T.) and siNEG-treated cells. Data are presented as mean ± SD from N=4 independent experiments. Statistical analysis was performed using Kruskal-Wallis followed by Dunn’s multiple comparisons test *versus* N.T. (**A**, **B**); ns >0.05, * <0.05. Analysis of TAZ expression following siYAP1 treatment and YAP1 expression following siTAZ treatment showed no significant differences compared to N.T. (data not annotated on the graph for clarity).

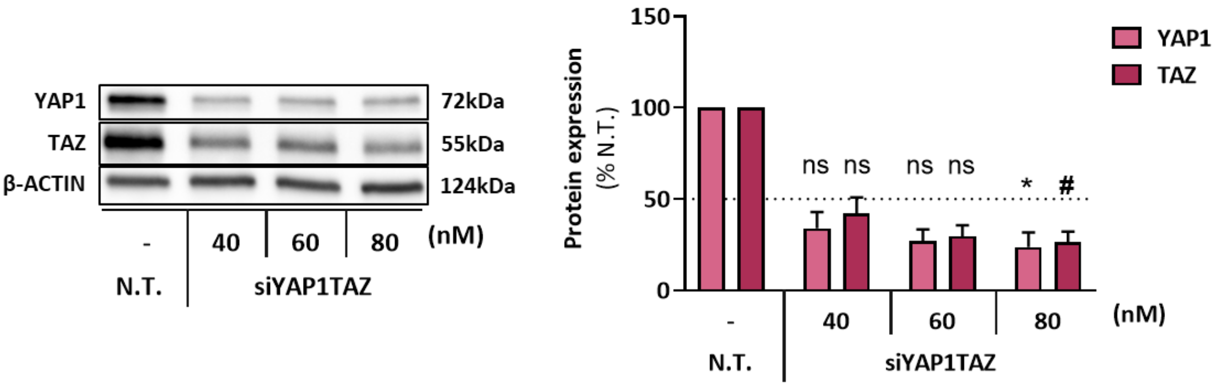

Figure S3: Silencing YAP1 and TAZ protein expression using a single siRNA in GIST-T1 cells
WRAP5-based nanoparticles encapsulating a single siRNA (siYAP1TAZ) published by Tiffon et al., 2020, induced a dose-dependent inhibition of YAP1 and TAZ simultaneously in GIST-T1 cells after 48 h of incubation, as shown by Western blot quantification.
Values are the mean ± SD for N=3 independent experiments. Statistical test: Kruskal-Wallis followed by Dunn with ns >0.05 and * or # <0.05 comparisons test *versus* the corresponding N.T. condition.

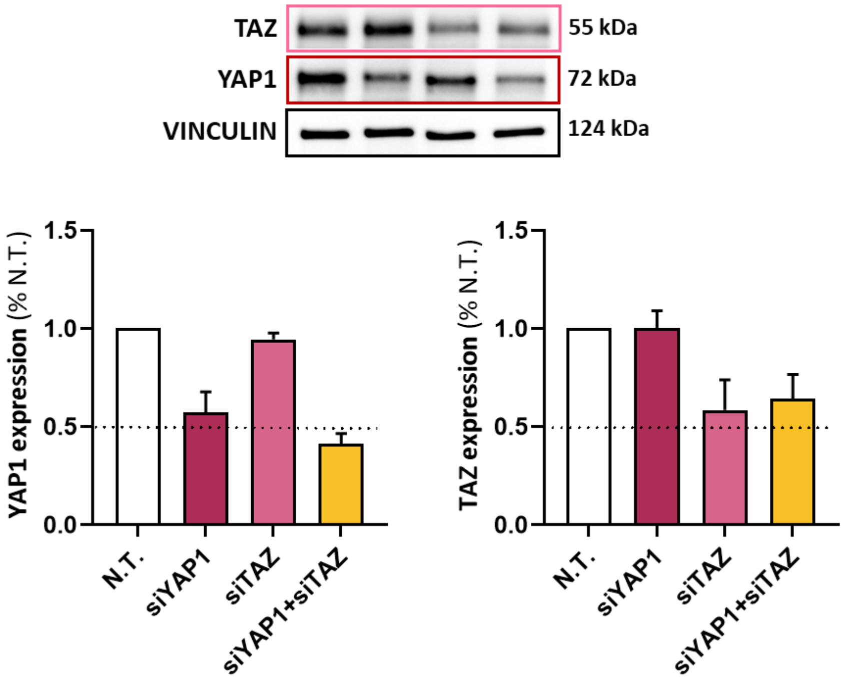

Figure S4: YAP1 and TAZ silencing persists over a period of 72 h in GIST-T1 cells
WRAP5:siRNA nanoparticles delivering siYAP1, siTAZ or both (siYAP1+siTAZ) induced inhibition of YAP1 or TAZ compared to non-treated cells (N.T.) in GIST-T1 cells after 72 h of incubation, as shown by Western blot quantification.
Data are presented as mean ± SD from N=2.

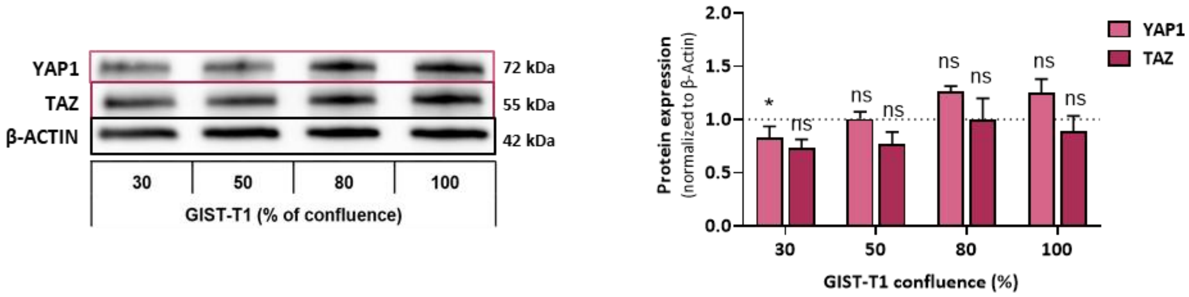

Figure S5 : YAP1 and TAZ protein expression in GIST-T1 cells

Western blot analysis and quantification of YAP1 and TAZ protein expression in GIST-T1 cells according to cell confluence. Values are the mean ± SEM for N=4 independent experiments. Statistical test: Kruskal-Wallis followed by Dunn with a multicomparison showing ns >0.05 and * <0.05 for YAP1 or TAZ expression at different confluences.

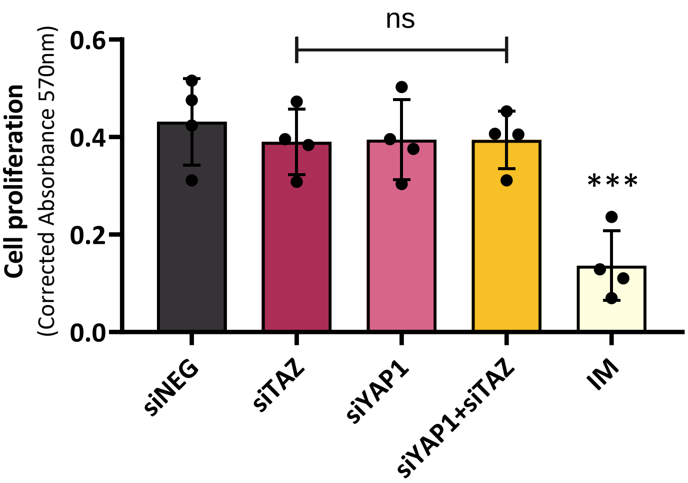

Figure S6: Cell proliferation remains unaffected at 48 h post siRNA transfection

Proliferation of GIST-T1 cells was assessed 48 h after transfection using the CellTiter 96® Non-Radioactive Cell Proliferation Assay for N=4 with n=5 each.

siNEG was used as a control. Abbreviation: IM = Imatinib. Data are presented as mean ± SD for N=4 independent experiments. Statistical analysis was performed using one-way ANOVA followed by Dunnett's multiple comparisons test *versus* siNEG; ns > 0.05, *** < 0.001.

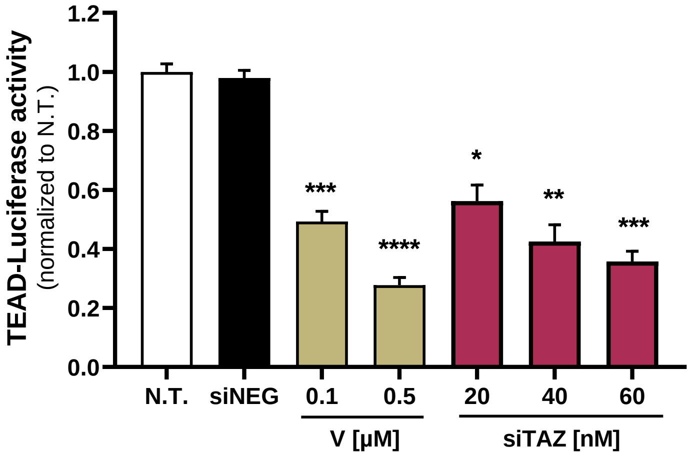

Figure S7: TAZ silencing prevents YAP1/TAZ-TEAD transcriptional activity.
YAP1/TAZ-TEAD transcriptional activity was assessed in GIST-T1 cells through the transfection of pTEAD-Luc/pRen or pTAL-Luc/pRen plasmids. After 6 h of plasmid transfection, the cells were incubated with WRAP5:siTAZ or with verteporfine (V) at the indicated concentrations. siNEG was used as a control. Data are presented as mean ± SEM for N=5 with n=3 each. Statistical analysis was performed using one-way ANOVA followed by Dunnett's multiple comparisons test *versus* siNEG; ns > 0.05, * <0.05, ** <0.01, *** < 0.001, and ****<0.0001.

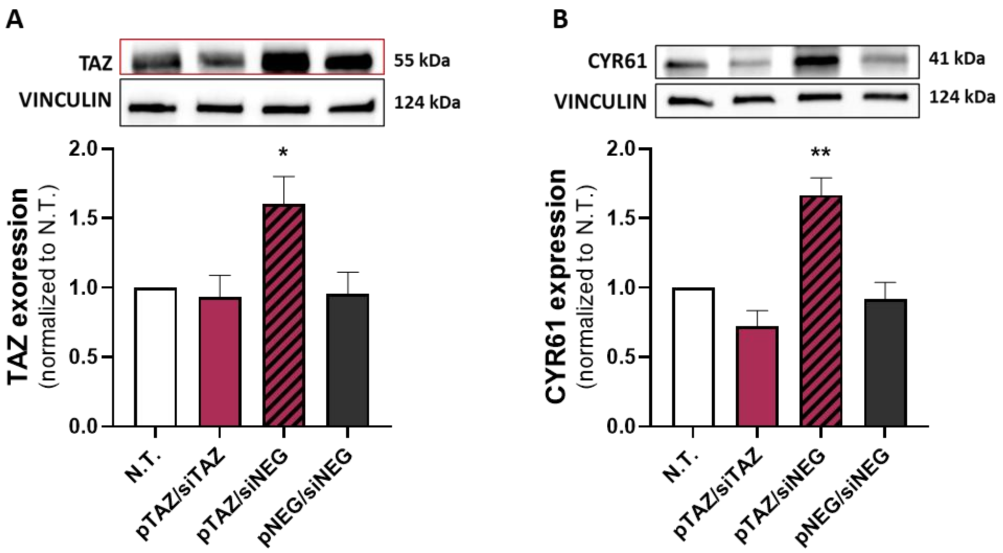

Figure S8: Evaluation of TAZ and CYR61 levels after TAZ overexpression in GIST-T1 cells
GIST-T1 cells were first transfected with a TAZ plasmid and then with WRAP5:siRNA nanoparticles delivering siTAZ and siNEG. After 48 h of incubation, levels of TAZ (**A**) or CYR61 (**B**) expression were quantified by Western blot analyses. Controls include untreated cells (N.T.) as well as pNEG/siNEG-treated cells (for more details, see the Supporting Method section above). Data are presented as mean ± SD from N=4. Statistical analysis was performed using one-way ANOVA followed by Dunnett's multiple comparisons test *versus* N.T. * <0.05, ** <0.01.

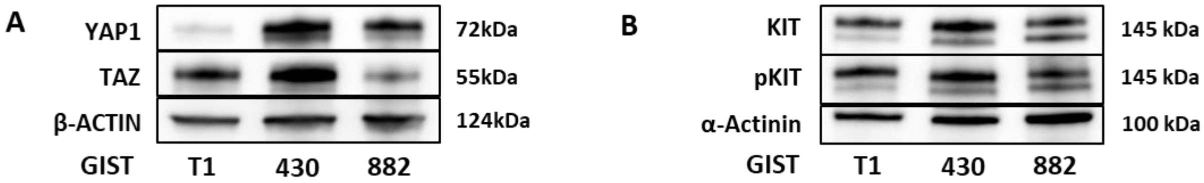

Figure S9: Basal expression of YAP1, TAZ, KIT, pKIT in GIST cell lines.
Comparison of the basal protein expression levels of YAP1 and TAZ (**A**) as well as KIT and phosphorylated KIT (pKIT) (**B**) in GIST-T1, GIST-430, and GIST-882 cell lines, as assessed by Western blot.

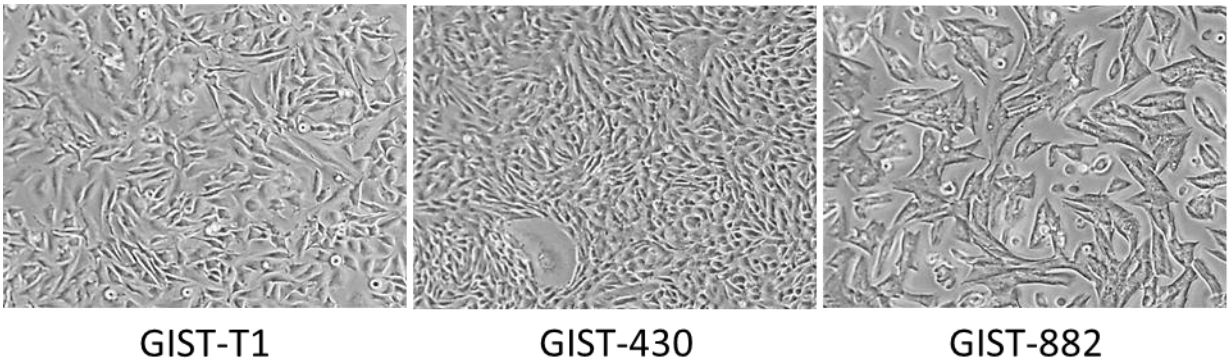

Figure S10: Morphology of three different GIST cell lines
GIST-T1, GIST-430, GIST-882 images taken using the EVOS® LX Core microscope system with a 10x objective.

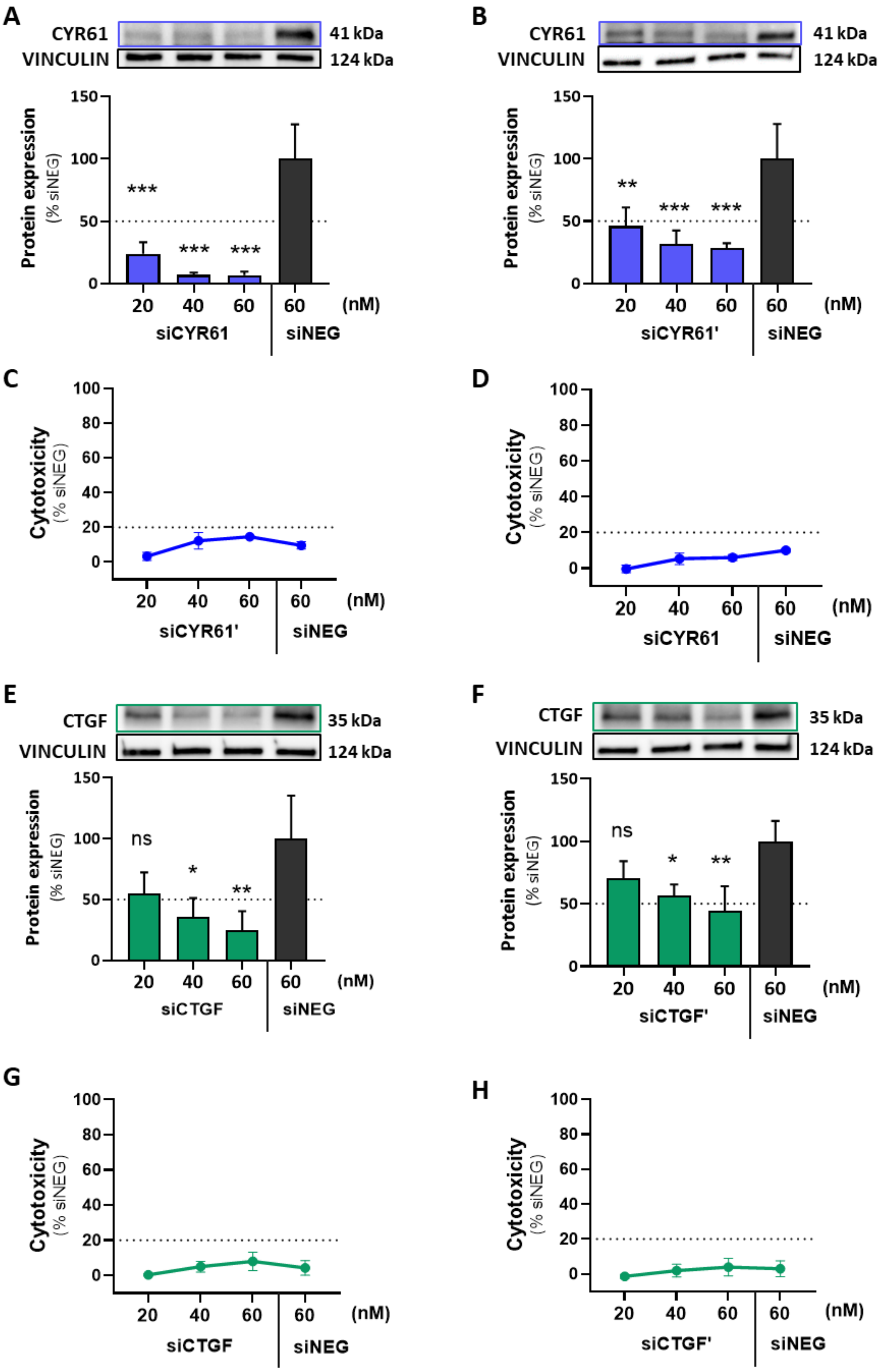

Figure S11: Evaluation of CYR61 or CTGF silencing in GIST-T1 cells with WRAP5:siRNA nanoparticles

WRAP5:siRNA nanoparticles delivering siCYR61 and siCYR61' (**A,B**) or siCTGF and siCTGF' (**E,F**) induced a dose-dependent inhibition of CYR61 or CTGF in GIST-T1 cells after 48 h of incubation, as shown by Western blot quantification. No toxicity was observed respectively for siCYR61 and siCYR61' (**C,D**) and siCTGF and siCTGF' (**G,H**), as assessed by LDH assay. Controls include untreated (N.T.) and siNEG-treated cells. Data are presented as mean ± SD from N=4 independent experiments. Statistical analysis was performed using Kruskal-Wallis followed by Dunn's multiple comparisons test *versus* siNEG. (**A**, **B**); ns >0.05, * <0.05, ** <0.01, *** <0.001.

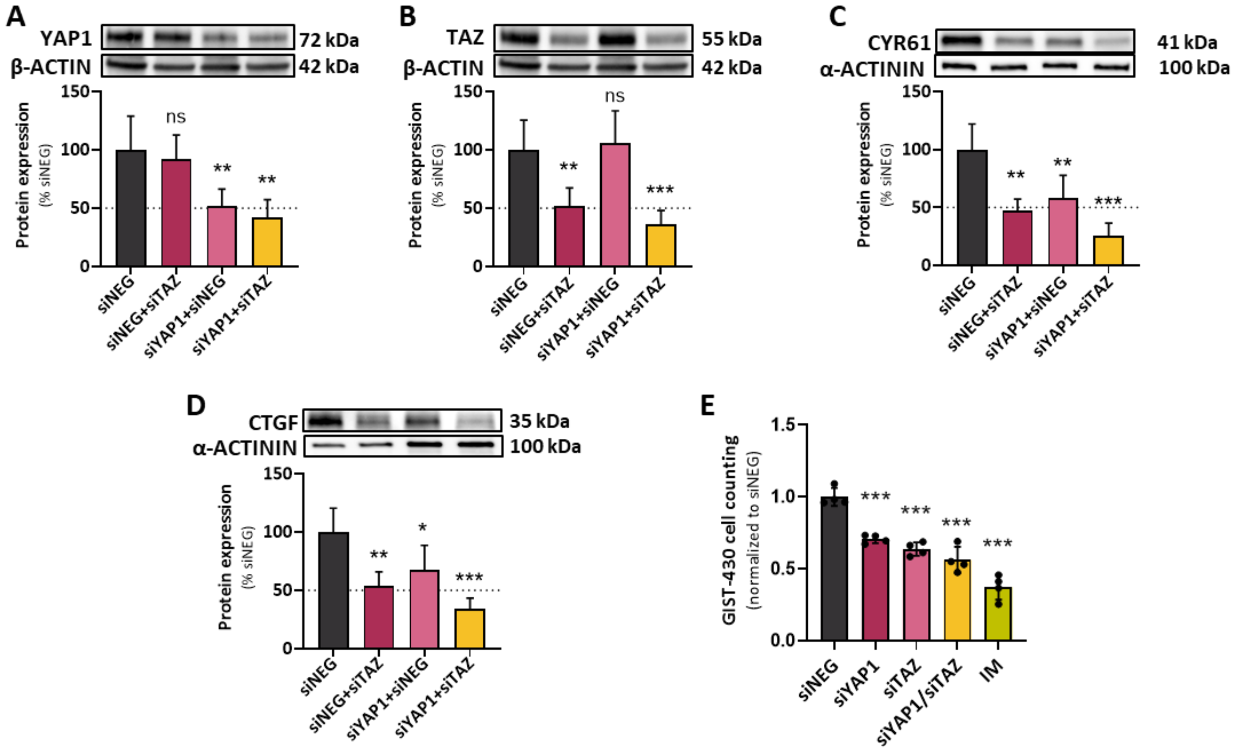

Figure S12: Effect of TAZ and YAP1 silencing in GIST-430 cells.
Effect of WRAP5:siRNA nanoparticles targeting TAZ, YAP1, or both (siYAP1+siTAZ) on YAP1 (**A**) and TAZ (**B**), as well as on CYR61 (**C**) and CTGF (**D**) protein expression in GIST-430 cells was analyzed by Western blot 48 h post-transfection (N=4-6 individual experiments).
(**E**) Effects of WRAP5:siRNA nanoparticles targeting TAZ, YAP1, or both (siYAP1+siTAZ) on proliferation were analyzed by cell counting, 72 h post-transfection in GIST-430 cells (N=4 with n=2 each).
Conditions: WRAP5:siRNA (MR 20 with [siRNA] = 80 nM), siNEG was used as a control. Data are presented as mean ± SD. Statistical analyses were performed using one-way ANOVA followed by Dunnett's post-hoc test *versus* siNEG (**A, B, C, D**), and one-way ANOVA followed by Holm-Sidak's post-hoc test *versus* siNEG (**E**), ns >0.05, * <0.05, ** <0.01, *** <0.001, **** <0.0001.

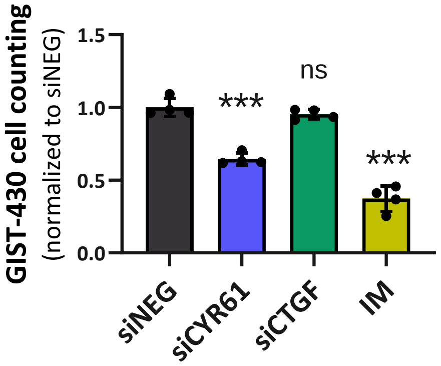

Figure S13: CYR61 is an effector of TAZ for GIST-430 cell proliferation.
WRAP5:siRNA nanoparticles targeting CYR61 or CTGF were analyzed by cell counting in GIST-T1 cells over a 72 h time course post-transfection in GIST-430 cells at 72 h post-transfection (N=4 with n=2 each).
Conditions: WRAP5:siRNA (MR 20) with [siRNA] = 20 nM for GIST-430 cells; siNEG was used as the control condition; IM (imatinib) = 50 nM. Data are presented as mean ± SD. Statistical analyses were performed using one-way ANOVA followed by Dunnett's post-hoc test *versus* siNEG (**E**); ns >0.05, *** <0.001.

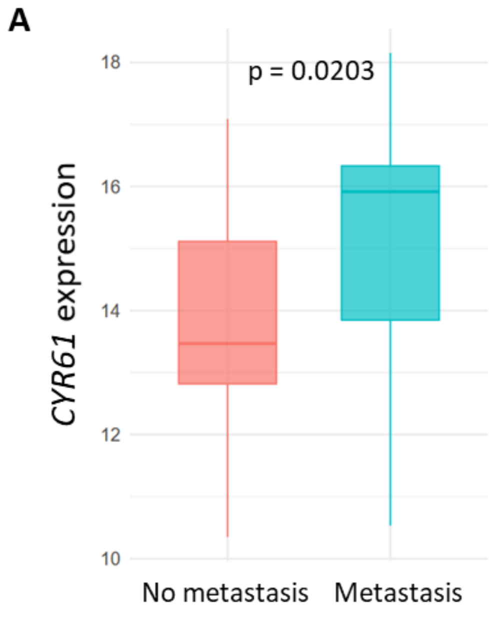

Figure S14: Correlation of *CYR61* and *CTGF* expression with the prognosis of GIST patients.
Correlation between *CYR61* or *CTGF* transcript levels and the presence of metastasis. n = 45 (red) and n = 15 (blue) in the non-metastatic and metastatic groups, respectively. p-values from the log-rank test are indicated.
